# Desmin mutations impact the autophagy flux in C2C12 cell in mutation-specific manner

**DOI:** 10.1007/s00441-023-03790-6

**Published:** 2023-06-06

**Authors:** K. S. Sukhareva, N. A. Smolina, A. I. Churkina, K. K. Kalugina, S. V. Zhuk, A. A. Khudiakov, A. A. Khodot, G. Faggian, G. B. Luciani, T. Sejersen, A. A. Kostareva

**Affiliations:** 1 Institute of Molecular Biology and Genetics, Almazov National Medical Research Centre, Saint-Petersburg, Russia; 2 Graduate School of Life and Health Science, University of Verona, Verona, Italy; 3 Department of Women’s and Children’s Health, Karolinska University Hospital, Karolinska Institutet, Stockholm, Sweden; 4 Department of Neuropaediatrics, Astrid Lindgren Children’s Hospital, Karolinska University Hospital, Stockholm, Sweden

**Keywords:** Desmin mutation, C2C12, Autophagy, BAG3, CASA, Protein aggregates

## Abstract

Desmin is the main intermediate filament of striated and smooth muscle cells and plays a crucial role in maintaining the stability of muscle fiber during contraction and relaxation cycles. Being a component of Z-disk area, desmin integrates autophagic pathways, and the disturbance of Z-disk proteins’ structure negatively affects chaperone-assisted selective autophagy (CASA). In the present study, we focused on alteration of autophagy flux in myoblasts expressing various *Des* mutations. We applied Western blotting, immunocytochemistry, RNA sequencing, and shRNA approach to demonstrate that *Des*S12F, *Des*A357P, *Des*L345P, *Des*L370P, and *Des*D399Y mutations. Mutation-specific effect on autophagy flux being most severe in aggregate-prone *Des* mutations such as *Des*L345P, *Des*L370P, and *Des*D399Y. RNA sequencing data confirmed the most prominent effect of these mutations on expression profile and, in particular, on autophagy-related genes. To verify CASA contribution to desmin aggregate formation, we suppressed CASA by knocking down *Bag3* and demonstrated that it promoted aggregate formation and lead to downregulation of *Vdac2* and *Vps4a* and upregulation of *Lamp*, *Pink1*, and *Prkn*. In conclusion, *Des* mutations showed a mutation-specific effect on autophagy flux in C2C12 cells with either a predominant impact on autophagosome maturation or on degradation and recycling processes. Aggregate-prone desmin mutations lead to the activation of basal autophagy level while suppressing the CASA pathway by knocking down *Bag3* can promote desmin aggregate formation.

**Supplementary Information:**

The online version contains supplementary material available at 10.1007/s00441-023-03790-6.

## Introduction

Being a classical representative of the intermediate filament system in muscle cells, desmin supports the mechanical stability of cardiomyocytes and skeletal muscle fibers under the constant mechanical load. Desmin filaments form a three-dimensional scaffold that ensures cellular compartmentalization and provides a transportation network for signaling pathways ((Capetanaki et al. 2007); (Schroder and Schoser 2009)). This scaffold is organized around the Z-disk area of myofilaments, thus allowing the control of the alliance of fibrils during contraction and relaxation cycles. In addition, this network provides the interaction of myofibrils with sarcolemma, nuclei, and membrane organelles, thus integrating the mechanotransduction process.

Mutations in the *DES* gene are associated with various muscular and cardiac pathologies, so-called desmin-related myopathies or cardiomyopathies. Histologically, these pathologies are often characterized by the presence of myofibrillar protein aggregates in muscle tissue, the main diagnostic criteria for myofibrillar myopathies ((Thornell et al. 1980); (Goldfarb et al. 2008); (Brodehl et al. 2018)). The presence of desmin aggregates in muscle cells causes the loss of contractility force, disorganization of the Z-disk region, and impairment of signaling pathways. The nature of these aggregates has been in focus of many cellular, biochemical, and molecular studies ((Perng et al. 2004); (Clemen et al. 2013); (Kedia et al. 2019); (Smolina et al. 2020)). Nowadays, it is generally accepted that these protein aggregates are composed of filamin A/C, sequestosome 1 (SQSTM1/p62), Xin actin-binding repeat-containing protein 1 (XIRP1), αB-crystallin, desmin, nestin, and several other proteins ((Feldkirchner et al. 2012); (Maerkens et al. 2016); (Singh et al. 2020)). Not all *DES* mutations result in aggregate formation. The ability to form the aggregates depends on the mutation site, its projection to the protein structure, and individual effects on the protein polymerization ((Bar et al. 2005); (Kostareva et al. 2011)). One of the most deleterious *DES* mutations, *DES*L345P located in the 2B part of the protein rod domain, resulted in marked loss of polymerization ability, formation of protein aggregates, violation of interaction with cell components, and the disruption of Z-disk regions in myofilaments ((Sjoberg et al. 1999); (Schroder et al. 2003)). Other *DES* mutations found in the rod domain, *DES*A357P, *DES*L370P, and *DES*D399Y, also form protein aggregates and are associated with severe cases of myofibrillar myopathy and cardiomyopathy ((Dagvadorj et al. 2003); (Arias et al. 2006); (Even et al. 2017)). On the other hand, mutations located in the desmin head or tail domains usually are not associated with aggregate formation while still lead to the development of cardiac and skeletal muscle pathology, for example, *DES*S12F ((Hong et al. 2009).

Myofibrillar structural proteins are subjected to a substantial mechanical load during the force transmission across the muscle cell. Protecting the muscle fiber components from damage and maintenance of proper protein folding is critical for the cellular integrity, myofilament mechanical strength, and functional activity of the cell. Therefore, autophagy is crucial for the on-time elimination of damaged cellular components, organelles, and aggregated proteins, and normally serves as a housekeeping process aimed at maintaining cellular homeostasis (Levine and Klionsky 2004). In addition, when cells are exposed to stress, such as starvation, autophagy becomes one of the key mechanisms of cellular survival by providing nutrients for cells ((Scott et al. 2004); (Nishida et al. 2008)).

There are several main types of autophagy: macroautophagy, microautophagy, and chaperon-mediated autophagy (Kaminskyy et al. 2011). In muscle cells, a subtype of macroautophaty—chaperone-assisted selective autophagy (CASA)—plays an important role, as this machinery helps to degrade damaged Z-line-associated proteins, thus supporting Z-disk integrity and proper organization of the cell. The CASA protein complex comprises the following proteins: HSPA8, HSPB8, HSP70, STUB1, SQSTM/p62, and BAG3 ((Behl 2011); (Ulbricht et al. 2013)). The latter is also a well-known protein associated with myofibrillar myopathies and cardiomyopathies where BAG3 deficiency can substantially deteriorate the autophagy process and protein aggregate formation ((Ji et al. 2019); (Adriaenssens et al. 2020); (Ruparelia et al. 2021)).

In muscle cells, *BAG3* expression is induced by different stimuli, and BAG3 is responsible for the formation of polyglutamine repeats before induction of target protein degradation through CASA ((Carra et al. 2008); (Gamerdinger et al. 2009); (Zhao et al. 2019)). In the Bag3-deficient mice model and in human cell cultures with *BAG3* mutations, the autophagy process was severely affected and thus resulting in the accumulation of α-actinin, myosin heavy chain, desmin, and vinculin in insoluble protein fraction, which proves the key role of BAG3 in the elimination of damaged proteins from muscle cells ((Fang et al. 2017); (Meister-Broekema et al. 2018)).

Although both aggregate formation and disturbed autophagy are well-known characteristics of desmin-related myopathies and cardiomyopathies, knowledge has been lacking regarding the role of autophagy in the molecular pathogenesis of desmin-related muscle pathology and aggregate formation. In the present study, we focused on autophagy flux in myoblasts expressing various *Des* mutations. We applied Western blotting to measure levels of autophagy-related proteins, immunocytochemistry to quantify autophagosome puncta in cells, RNA sequencing to determine gene expression profiles, and shRNA approach to promote aggregate accumulation.

We demonstrated that alteration of autophagy flux has a mutation-specific pattern, being the most overt in aggregate-prone *Des* mutations. Also, we showed that knock-down of *Bag3* impaired autophagy and potentiate the formation of desmin aggregates, therefore underscoring the role of autophagy in the accumulation of desmin aggregates.

## Materials and methods

### Cell culture

The work was performed on mouse immortalized myoblasts C2C12 (ATCC, USA, catalog number: CRL 1772). Cells were cultivated in DMEM medium (Gibco, USA) supplemented with 10% fetal bovine serum (FBS) (Gibco, USA), 2 mM L-glutamine, 50 μg/ml penicillin, and 50 μg/ml streptomycin. Cells were incubated at 37 °C, 5% CO2, and 99% humidity and were re-plated every 72 h in the amount of 100,000 on a 100 mm Petri dish.

### The experimental design

C2C12 cells were transduced with lentiviruses (LV) carrying *Des* wild-type (*Des*WT) or mutant *Des* (*Des*L345P, *Des*A357P, *Des*L370P, *Des*D399Y, *Des*S12F). After 72 h of LV transduction, starvation-induced autophagy was triggered by incubating cells in EBSS (Sigma, Germany) for 2–8 h. Western blotting was used to evaluate the ratio of LC3 protein isoforms: LC3-I–a soluble or free protein fraction in the cytoplasm and LC3-II–a fraction bound or integrated into the autophagosome membrane, immunocytochemistry was applied to quantify LC3 puncta within the cells, while qPCR was used to evaluate the gene expression of proteins involved in autophagy.

### Cultivation and viral transduction of C2C12 cells

LV were produced in HEK293T cells. Cells were transfected with pLeGO-SFFV/IRES-eGFP (20 μg) that expressed *Des*WT, or *Des*L345P, *Des*A357P, *Des*L370P, *Des*D399Y, *Des*S12F, with pMD2.G (5 μg) and psPAX2 (5 μg). Wild-type desmin was subcloned in pLeGO using EcoRI-NotI restriction site. Plasmids with desired mutation were obtained as described in using pLeGO-SFFV/IRES-eGFP (Smolina et al. 2020).To increase the efficiency of transduction, PEI reagent (Sigma, USA) was added to the culture medium at the ratio of 2:1 per total amount of pDNA. The concentration of obtained viruses was performed as described previously (Smolina et al. 2020). The expression of exogenous *Des* in C2C12 cell was performed using LV transduction with 20 μl of concentrated viral suspension per 60,000 cells in the presence of Polybrene (Sigma, Germany) at a concentration of 8 μg/ml for the entire volume of the growth medium.

### Stimulation of the autophagy process

After transduction with *Des*WT and *Des*L345P, *Des*A357P, *Des*L370P, *Des*D399Y, and *Des*S12F, cells were plated at a density of 30,000 or 50,000 cells per 60-mm plate for subsequent detection by immunocytochemistry or Western blotting, respectively. Seventy-two hours after the transduction, the growth medium was replaced with EBSS to induce autophagy. Observation of autophagy was carried out 2–8 h after induction. The inhibitor of autophagosome and lysosome fusion chloroquine (CQ) was applied at a concentration of 100 μM at the time point of 2 h both to experimental and control samples.

### Western blotting analysis

To characterize autophagy flux, we performed a quantitative analysis of LC3 protein isoforms, using a protocol developed earlier for C2C12 cells ((Kaminskyy et al. 2011); (Klionsky et al. 2016); Sukhareva et al. 2016). After autophagy stimulation, the cells were collected in a sample tube, pelleted by centrifugation at 1000 g for 5 min. Permeabilization of cellular sediment was performed using the 0.025% digitonin (Sigma, USA) in PBS for 5 min on ice. After centrifugation at 2000 g for 5 min, supernatant was collected for further analysis of the soluble protein isoform LC3 (LC3-I). Extraction of the insoluble fraction of the LC3 protein (LC3-II) was performed by lysing cell sediment in RIPA buffer containing a cocktail of protease inhibitors (Roche, USA) and modification of several components to increase the effectivity of the lysis (1% NP-40, 0.5% sodium deoxycholate, 1% SDS, 1% Triton-X 100, 5 mM EDTA) for 10 min on ice. The protein lysates were centrifuged at 16,000 g for 10 min. The resultant supernatant was collected in a new test tube for further analysis of the insoluble LC3-II isoform associated with the autophagosome membrane. Laemmli buffer was added to all protein lysates and further incubated for 5 min at 100 °C.

Protein lysates were run in 12.5% polyacrylamide gel at a constant current of 20 mA in the stacking gel and 40 mA in the resolving gel. Separated proteins were transferred to a nitrocellulose membrane (Applichem, USA) with a pore diameter of 0.45 microns for 1 h at a constant voltage of 100 V using a transfer buffer (49.9 mM TrisHCl, 38.6 mM glycine, 0.0385% SDS, 20% methanol). To assess the quality of the protein transfer, the membranes were reversibly stained with Ponceau S dye (Sigma-Aldrich, USA), followed by washing in PBS with 0.05% Tween 20 (PBS-T). Non-specific antibody binding was blocked with 5% milk diluted in PBS-T for 30 min at room temperature (RT). The membranes were incubated with primary polyclonal anti-LC3 antibody (MBL International Corporation, USA) and with primary anti-alpha-actinin (Agilent, USA) in a 1:7000 and 1:200 dilutions, respectively, in 5% milk in PBS-T overnight at 4 °C. The membranes were washed 3 times in PBS-T and then incubated with a secondary anti-rabbit antibody, conjugated with horseradish peroxidase (BioRad, USA) at a concentration of 1:20,000 in PBS-T for 1 h at RT. The membranes were developed with SuperSignal West Femto substrate (Thermo Fisher Scientific) and processed in the Vilber Lourmat luminescent signal detection chamber (BioRad, USA). Data obtained by Western blotting were processed using the quantification analysis module of Fusion software. The evaluation of the amount of protein in the band was performed by estimating the optical density of pixels in the selected area of the sample. Total protein levels across the different lanes were normalized by analyzing Ponceau S staining of the membranes (Sigma-Aldrich, USA). Protein level for each mutation sample from each experiment was evaluated relative to its control sample. Each control sample was assigned to 1.0.

### Immunocytochemistry

Evaluation of LC3-positive puncta within the cells was performed by immunocytochemical staining with an anti-LC3 antibody. Cells seeded on coverslips were subjected to starvation for 2 h. After starvation, the cell membrane was permeabilized with 0.005% digitonin in PBS for 5 min on ice without prefixation to eliminate the soluble fraction of LC3 protein. Prior to the fixation, cells were washed 3 times in PBS for 5 min. Cells were fixed with 4% paraformaldehyde (Sigma, USA) for 5 min on ice and then blocked with 15% FCS in PBS at RT for 30 min. Incubation with the primary polyclonal anti-LC3 antibody at a dilution of 1:500 was performed at RT for 1 h. Goat anti-rabbit secondary antibody (Alexa Fluor 560, Thermo Fisher Scientific, USA) in a dilution of 1:200 was applied to the cells for 45 min at RT in the dark. DAPI (Invitrogen, USA) was used for 30 s to counterstain nuclei. Quantification of LC3-II signal in cells after immunocytochemical staining was performed by counting the positively stained puncta on the images captured with the Observer.D1 fluorescent microscope in the MosaiX program (Carl Zeiss Microsystems, Germany).

### Cell survival assay

To verify cells viability latter were trypsinized from the cultivation dish and stained with Propidium Iodide (Sigma-Aldrich, MO, USA) for 20 min in the dark using the manufacturer’s recommendations. Flow cytometry was performed using the Cytoflex S flow cytometer (Beckman Coulter, California, USA) by determining the relative percentages of positive (apoptosis/necrosis) and negative (living) cells, initially logically gated using forward and side light scattering.

### RNA sequencing and gene expression analysis

Total RNA for RNA library preparation and sequencing was extracted using ExtractRNA reagent (Evrogen, Russia) according to the manufacturer’s instructions. Extracted RNA was quantified using Qubit (ThermoFisher, USA), the integrity of RNA was assessed in 1.5% agarose gel. RNA libraries were prepared in accordance with the manufacturer’s reference guide using TruSeq Stranded mRNA kit (Illumina, USA) with dual TruSeq CD Indexes (Illumina, USA). The final libraries’ concentrations and quality were evaluated by Bioanalyzer 2100 (Agilent, USA) using HS DNA chip. Sequencing was performed on the NextSeq 2000.

Bcl files obtained after RNA sequencing were converted into fastq format using the bcl2fastq conversion software v1.8.4 (Illumina).

For RNA-seq data processing, nf-core/rnaseq pipeline was used (Harshil Patel et al. 2022). Briefly, paired-end reads were trimmed using TrimGalore v0.6.7, ribosomal RNA sequences were removed with SortMeRNA v4.3.4, reads were aligned to GRCm38 genome with STAR v2.7.10a, deduplication was performed with Picard v2.25.0 MarkDuplicates, and transcripts were quantified using RSEM v1.3.3.

After the counts data were obtained, DESeq2 (Love, 2014) was used to perform differential expression analysis. C2C12 cells with wild-type desmin (*Des*WT) were compared with samples with mutant desmin (*Des*L345P, *Des*A357P, *Des*L370P, *Des*D399Y, and *Des*S12F) to determine the effect of *Des* mutations pretense on basal gene expression. All samples on scr*Bag3* background were compared to all samples on sh*Bag3* background to evaluate the sh*Bag3* effect. Samples with *Des*WT on sh*Bag3* background were compared with sh*Bag3* background and mutant desmin (*Des*L345P, *Des*A357P, *Des*L370P, *Des*D399Y, and *Des*S12F). Phantasus was used to create heat maps for gene sets off interest as a web application for visual and interactive analysis of gene expression (https://artyomovlab.wustl.edu/phantasus/).

For real-time PCR analysis, total RNA was extracted as described above and quantified with NanoDrop 3300 (ThermoFisher, USA). Complementary DNA (cDNA) was synthesized using random primers and MMLV reverse transcription kit (Evrogen, Russia). Gene expression was quantified using qPCR with qPCR mix-HS SYBR + ROX (Evrogen, Russia). Sequences for primers are listed in Supplementary Table 1. The expression level of *Gapdh* was used to normalize the changes in the expression of genes of interest. For the relative quantification of gene expression, RQ = 2^(−∆∆Ct)^ was calculated and used to compare gene expression in studied samples.

### Data processing, visualization, and statistics

The GraphPad program was used for statistical data processing and plotting. The results obtained are presented as an average value for all performed experiments. The reliability of differences between groups was assessed using the non-parametric Mann-Whitney test. Differences were considered significant at the level of P < 0.05.

## Results

### Viral transduction increases basal autophagy level in C2C12 cells

**Fig. 1:**
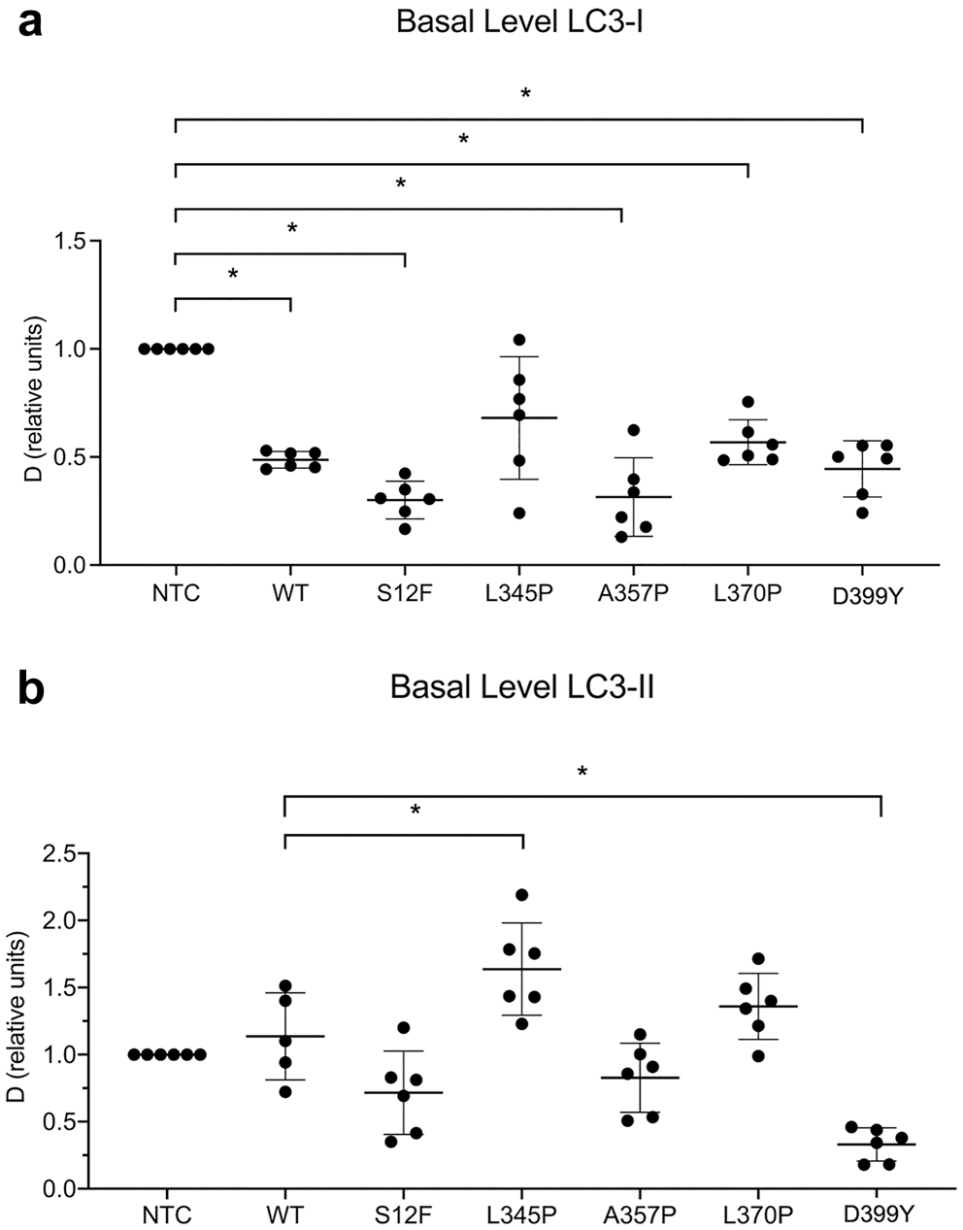
Basal autophagy level shown by the LC3-I and LC3-II dynamics in NTC C2C12 cells and C2C12 with *Des*WT, *Des*S12F, *Des*L345P, *Des*A357P, *Des*L370P, and *Des*D399 mutations. **a** LC3-I protein dynamics. **b** LC3-II protein dynamics. * < 0.05. Data presented as mean +—SD. *n* = 6 biological replicates

To evaluate the effect of viral transduction and *Des* mutations on autophagy flux in C2C12 cells, we assessed the basal LC3-I and LC3-II levels without stimulation. LC3-I and LC3-II were measured separately after transduction with LV carrying *Des*WT and *Des* mutations *Des*S12F, *Des*L345P, *Des*A357P, *Des*L370P, and *Des*D399Y and compared to NTC cells (Fig. 1). In *Des*WT and mutation samples, LC3-I levels were reduced almost by half compared to NTC cells with no significant difference between WT and mutant *Des* (Fig. 1a). Thus, LV transduction per se led to LC3-I decrease regardless of the type of LV-encoded exogenous *Des*. The amount of LC3-II after LV transduction with *Des* WT was not changed significantly compared to NTC (Fig. 1b). However, transduction with *Des*L345P resulted in an increase of LC3-II compared to WT cells. On the other hand, transduction with *Des*D399Y resulted in a drastic decrease of LC3-II.

Thus, LV transduction boosted autophagy in all samples. However, the further processing of LC3 and the amount of conjugated LC3-II isoform depended on autophagosome formation and degradation rates and was modulated in a mutation-specific manner.

### Autophagy stimulation under various desmin mutations demonstrates a mutation-specific response

According to our the previous study for further experiments, we chose the earliest time point of 2 h post-stimulation as the most informative to assess the autophagy dynamics in C2C12 cells transduced with *Des* mutations (Sukhareva et al. 2021). We considered later time points less informative due to the less striking difference between WT and mutant cells. Moreover, the extended periods of autophagy stimulation negatively affected cell viability due to the high toxic effect of both viral transduction and serum deprivation.

**Fig. 2:**
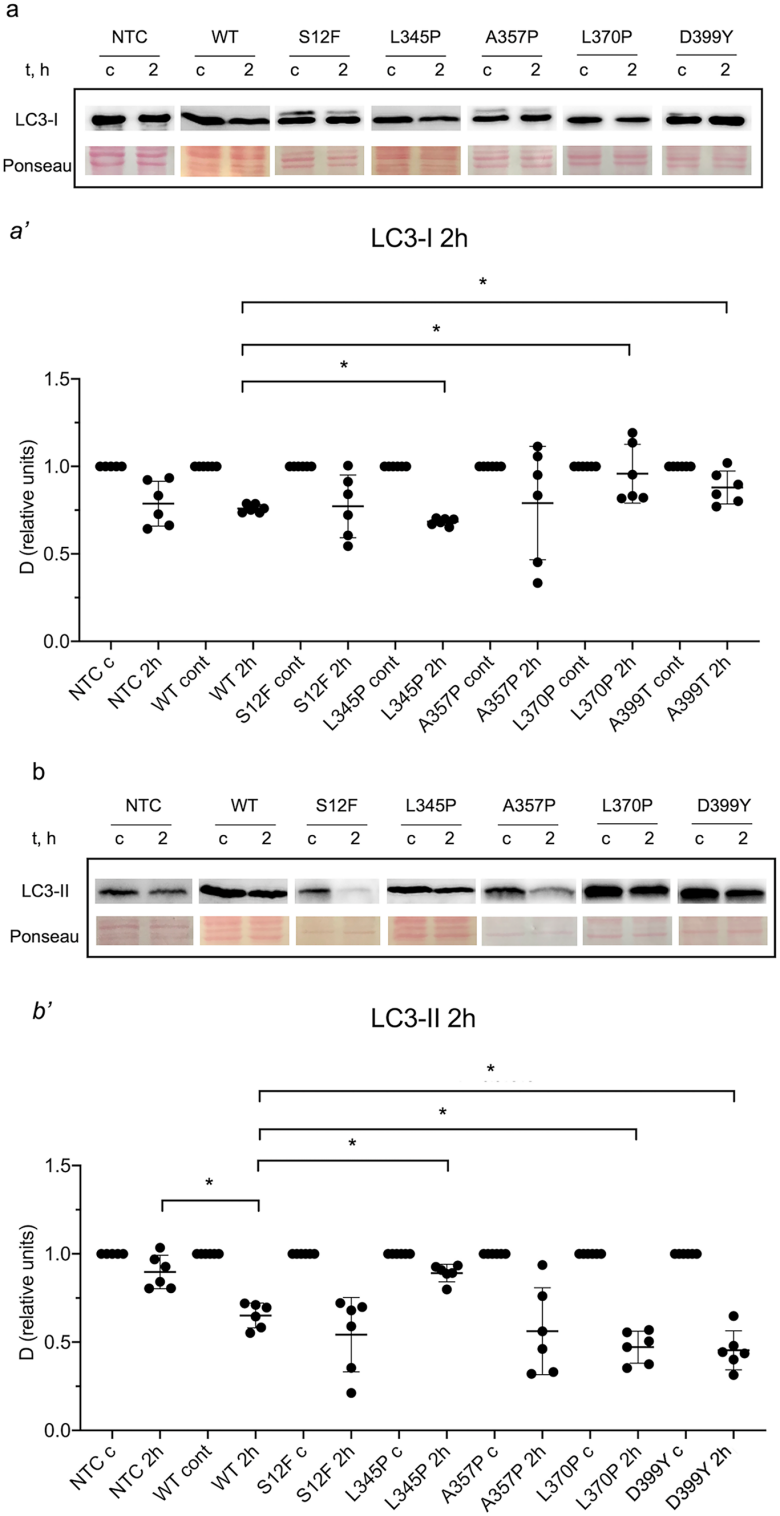
Autophagy dynamics in NTC C2C12 cells and C2C12 carrying various *Des* mutations: *Des*WT, *Des*S12F, *Des*L345P, *Des*A357P, *Des*L370P, *Des*D399Y, shown by the **a′** LC3-I and **b′** LC3-II isoform dynamics in control samples and after 2 h of starvations. **a** and **b** Western blotting membranes stained with LC3 antibody to visualize LC3 isoforms.**(a′, b′)** Graph representation of LC3 isoforms dynamics. * < 0.05. Data presented as mean +—SD. *n* = 6 biological replicates

To determine the impact of *Des* mutations on autophagy flux in C2C12 cells, autophagy was induced by starvation for 2 h after transduction with LV carrying *Des*WT and *Des* mutations *Des*S12F, *Des*L345P, *Des*A357P, *Des*L370P, and *Des*D399Y. LC3-I and LC3-II levels were evaluated separately, as previously described (Fig. 2). LC3-I level was similarly decreased in NTC, *Des*WT, *Des*S12F, and *Des*A357P cells after 2 h of starvation (Fig. 2(a, a′)). In cells carrying *Des*L345P after 2 h of serum deprivation, the level of LC3-I was significantly reduced compared to *Des*WT. The opposite tendency was observed for *Des*L370P and *Des*D399Y, where LC3-I was upregulated. Distinct trends were noticed in LC3-II dynamics. LV transduction per se reduced the level of LC3-II, as was observed by the difference between *Des*WT and NTC samples after 2 h of starvation (Fig. 2(b, b′)). No significant effect on LC3-II level was found in *Des*S12F and *Des*A357P compared to *Des*WT. However, the levels of LC3-II differed between *Des*WT and *Des*L345P as well as between *Des*WT and *Des*L370P and *Des*D399Y. The decline of LC3-II level after 2 h of starvation in *Des*L345P was significantly less pronounced compared to *Des*WT, when *Des*L370P and *Des*D399Y mutations caused a significantly more prominent decrease of LC3-II compared to *Des*WT.

In summary, the expression of *Des*L345P, *Des*L370P, and *Des*D399Y affected the level of stimulated autophagy and the amount of LC3-I and LC3-II in a mutation-dependent manner. The differences between the mutation effect can be attributed to the diverse stages of autophagosome maturation or degradation affected by the presence of mutations. Thus, *Des* mutations could control the autophagy flux in various ways, potentially due to the distinct mechanism underlying molecular effects of different *Des* mutations.

### Various desmin mutations impact autophagy by interfering distinct steps of autophagy

**Fig. 3:**
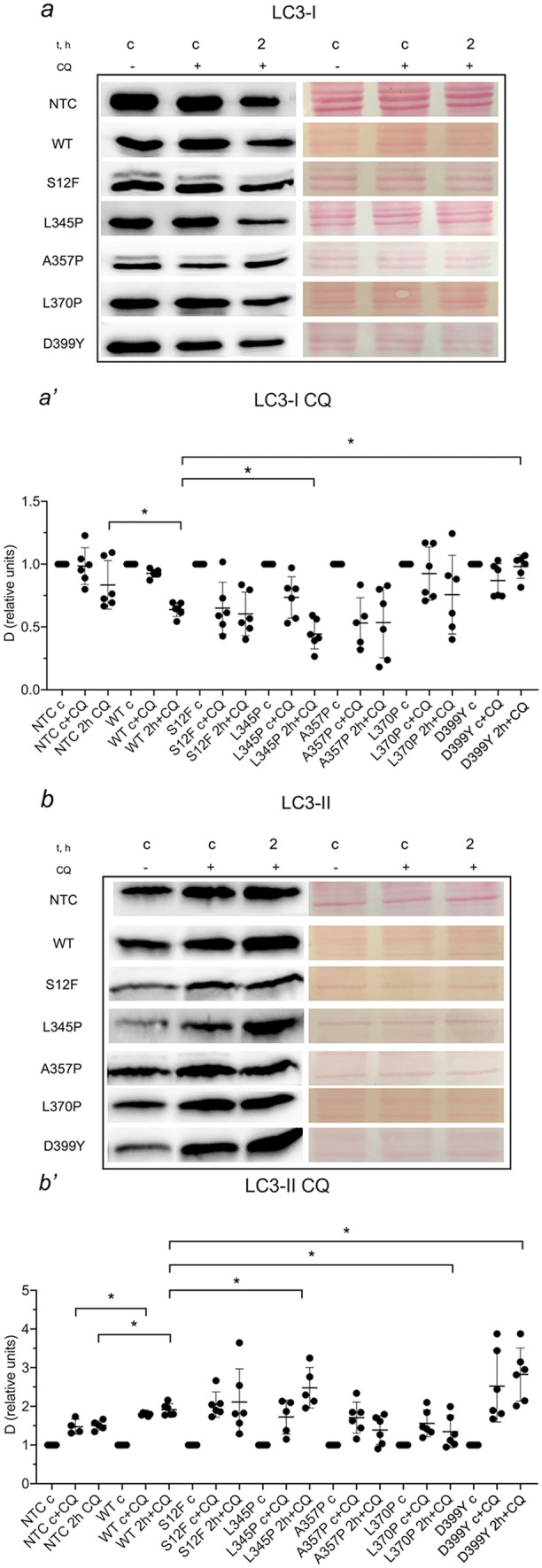
Autophagy dynamics in NTC C2C12 cells and C2C12 carrying various *Des* mutations: *Des*WT, *Des*S12F, *Des*L345P, *Des*A357P, *Des*L370P, *Des*D399Y, shown by the **a′** LC3-I and **b′** LC3-II isoform dynamics after 2 h of starvations and use of autophagy inhibitor chloroquine (CQ). **a **and **b** Western blotting membranes stained with LC3 antibody to visualize LC3 isoform. (**a′**, **b′**) Graph representation of LC3 isoforms dynamics. * < 0.05. Data presented as mean +—SD. *n* = 6 biological replicates

To decipher deeper the mechanisms of autophagy flux under the effect of *Des* mutations, we applied an inhibitor of autophagy CQ (chloroquine) for 2 h along with serum deprivation. Under CQ treatment, the level of LC3-I in *Des*WT demonstrated a more prominent decrease compared to NTC (Fig. 3(a, b)). In samples with *Des*S12F and *Des*A357P, a similar to *Des*WT decline of LC3-I level was observed, while in *Des*L345P, the decrease was significantly greater than in WT cells. In contrast, the LC3-I level under CQ treatment was almost unaltered in *Des*L370P and *Des*D399Y (Fig. 3(a, b)).

The level of LC3-II upon starvation under CQ treatment was increased in *Des*WT compared to CQ-treated NTC cells, meaning that cells with *Des*WT accumulated LC3-II more extensively (Fig. 3(a′, b′)). In *Des*S12F and *Des*A357P cells, LC3-II demonstrated similar to *Des*WT dynamics. In contrast, cells carrying *Des*L345P and *Des*D399Y accumulated LC3-II more efficiently than *Des*WT. Surprisingly, in *Des*L370P, LC3-II was slightly decreased after CQ treatment.

In summary, blockade of autophagy flux with autophagy inhibitor CQ allowed to estimate the stage of autophagy turnover modulated by *Des* mutations. Thus, *Des*L345P presumably causes the impairment of autolysosome processing and degradation, leading to marked accumulation of LC3-II. In contrast, *Des*L370P is likely associated with impaired LC3-I to LC3-II conversion leading to the under-processing of LC3-I and lack of LC3-II accumulation. In *Des*D399Y, the overall increase in the rate of autophagosome formation and autolysosomal degradation was observed which resulted in significant LC3-II accumulation under CQ treatment.

### Autophagy in C2C12 cells transduced with *Des* mutations after 2 h of stimulation assessed by immunocytochemistry

We performed the immunocytochemical staining to evaluate the number of LC3-positive puncta in C2C12 cells. In the first step, we assessed the basal level of autophagy in NTC cell and cells after LV transduction with *Des*WT and *Des* mutations: S12F, L345P, A357P, L370P, and D399Y (Supplementary Fig. 1). Immunostaining partially confirmed the results obtained by Western blotting. We found that the relative per mutation amount of autophagosomes in samples after LV transduction was higher compared to NTC cells. Moreover, the number of autophagosomes in *Des*L345P was the highest among the studied samples. However, we did not observe a significant difference for *Des*L345P and *Des*A357P after 2 h of serum deprivation, while the number of autophagosomes in *Des*S12F and *Des*L370P was greater compared to *Des*WT and lower in *Des*D399Y compared to *Des*WT. Thus, immunocytostaining results corroborated the data indicating that LV transduction increased the basal activity of autophagy.

### Aggregate-prone desmin mutations *Des*L345P, *Des*L370P, and *Des*D399Y are associated with alterations in cell expression profile

**Fig. 4:**
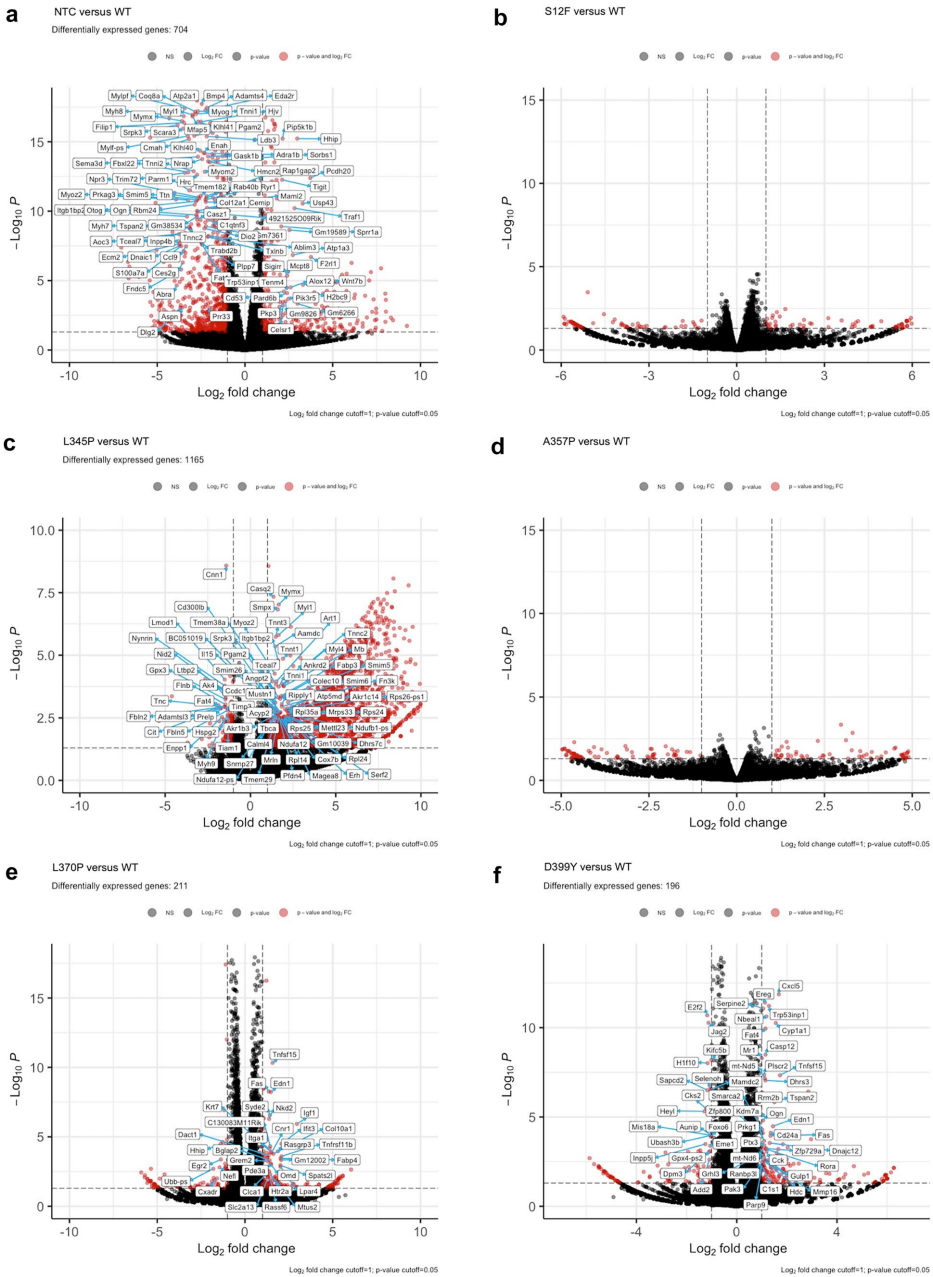
Gene expression analysis by RNA sequencing of C2C12 samples transuded with *Des* mutations: S12F, L345P. A357P, L370P, D399Y. (**a**, **b**, **c**, **d**, **e**, **f**) Volcano plot illustrations of RNA-seq differential expression data. Pairwise comparisons is shown between *Des*WT and the rest of mutations (S12F, L345P, A357P, L370P, and D399Y)

To determine whether a higher level of basal autophagy in the presence of *Des* mutations was associated with the upregulation of genes involved in autophagy pathways or solely caused by protein machinery activation, RNA sequencing was performed. The comparison of each *Des* mutation: *Des*S12F, *Des*L345P, *Des*A357P, *Des*L370P, and *Des*D399Y with *Des*WT revealed the following numbers of differentially expressed genes: 0, 1165, 0, 211, and 196 for each mutation, respectively (Fig. 4(a–f)). The highest number of differentially expressed genes was found for *Des*L345P, followed by *Des*L370P and *Des*D399Y samples. The absence of differentially expressed genes in *Des*S12F and *Des*A357P corresponded to Western blotting data, thus showing the major effect of *Des*L345P, *Des*L370P, and *Des*D399Y mutations on muscle cell function. Of note, *Des*WT cells differentially expressed 704 genes compared to NTC cells, which confirmed the significant impact of LV transduction on C2C12 expression profile.

**Fig. 5:**
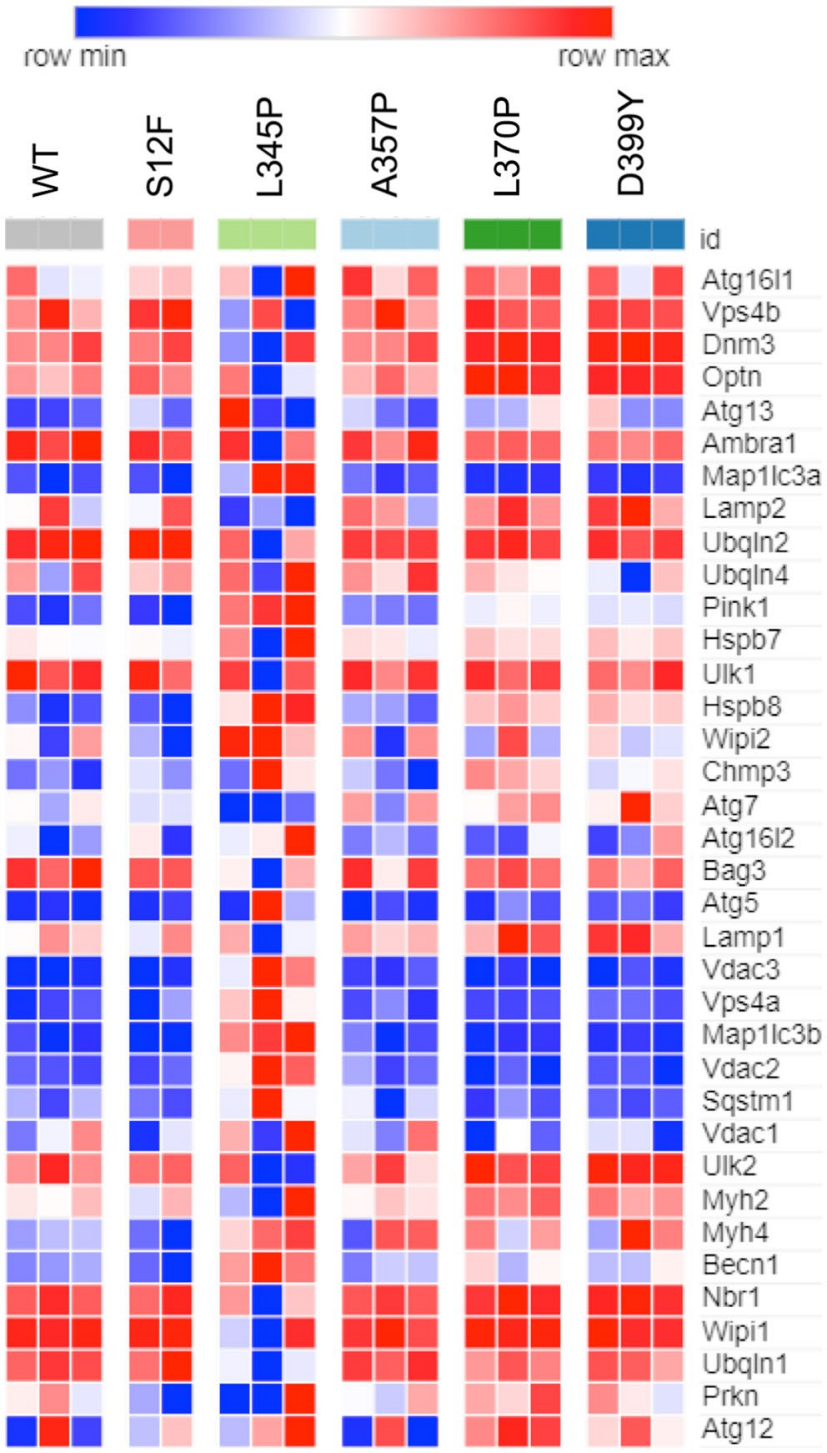
Gene expression analysis by RNA sequencing of C2C12 samples transuded with *Des* mutations: S12F, L345P. A357P, L370P, D399Y. Heat map illustrating genes associated with autophagy process. Pairwise comparisons is shown between *Des*WT with the rest of mutations (S12F, L345P, A357P, L370P, and D399Y). Blue, negative log fold-change (log FC) indicates lower expression; red, positive log FC

Albeit the autophagy pathway was not among the significantly upregulated ones, we performed a more detailed analysis and focused on the autophagy- and CASA-associated gene expression profiles in C2C12 cells after transduction with *Des* mutations. The count-based differential expression analysis and its visualization showed that some autophagy-related genes such as *Hspb7*, *Hspb8*, *Wipi2*, *Atg12*, *Chmp3*, and *Becn1* were highly upregulated in *Des*L345P, *Des*L370P, and *Des*D399Y samples comparing to *Des*WT (Fig. 5). Moreover, in *Des*L345P samples, there were several specifically upregulated genes: *Map1Lc3a*, *Map1Lc3b*, *Pink1*; and several specifically downregulated genes: *Vps4b*, *Lamp2*, *Atg7.* Expression of some genes in *Des*L370P and *Des*D399Y greatly differed from *Des*L345P; for instance, *Map1Lc3a*, *Map1Lc3b*, and *Becn1* were downregulated and *Vps4b*, *Dnm3*, *Optn*, *Ulk2*, and *Myh2* were upregulated. To verify RNA sequencing data, we performed qPCR and confirmed the upregulation of autophagy and CASA-related genes such as *Sqstm1*, *Bag3*, *Hspb7*, and *Hspb8* as well as upregulation of *Des* itself (Supplemental Fig. 2).

In summary, *Des*L345P, *Des*L370P, and *Des*D399Y demonstrated the most significant effect on gene expression, which corresponded to the Western blotting results and previously reported aggregate capacity of these mutations ((Bar et al. 2005)). Although the autophagy pathway was not among the ones significantly altered, the obtained data on gene regulation of autophagy in cells expressing *Des* mutations corresponded to Western blotting results on basal autophagy level demonstrating the prominent effect of LV transduction on gene expression profile and major changes in gene expression among samples with *Des*L345P, *Des*L370P, and *Des*D399Y mutations.

### *Bag3* suppression is associated with alteration of autophagy-related pathway and results in *Des* aggregate formation

**Fig. 6:**
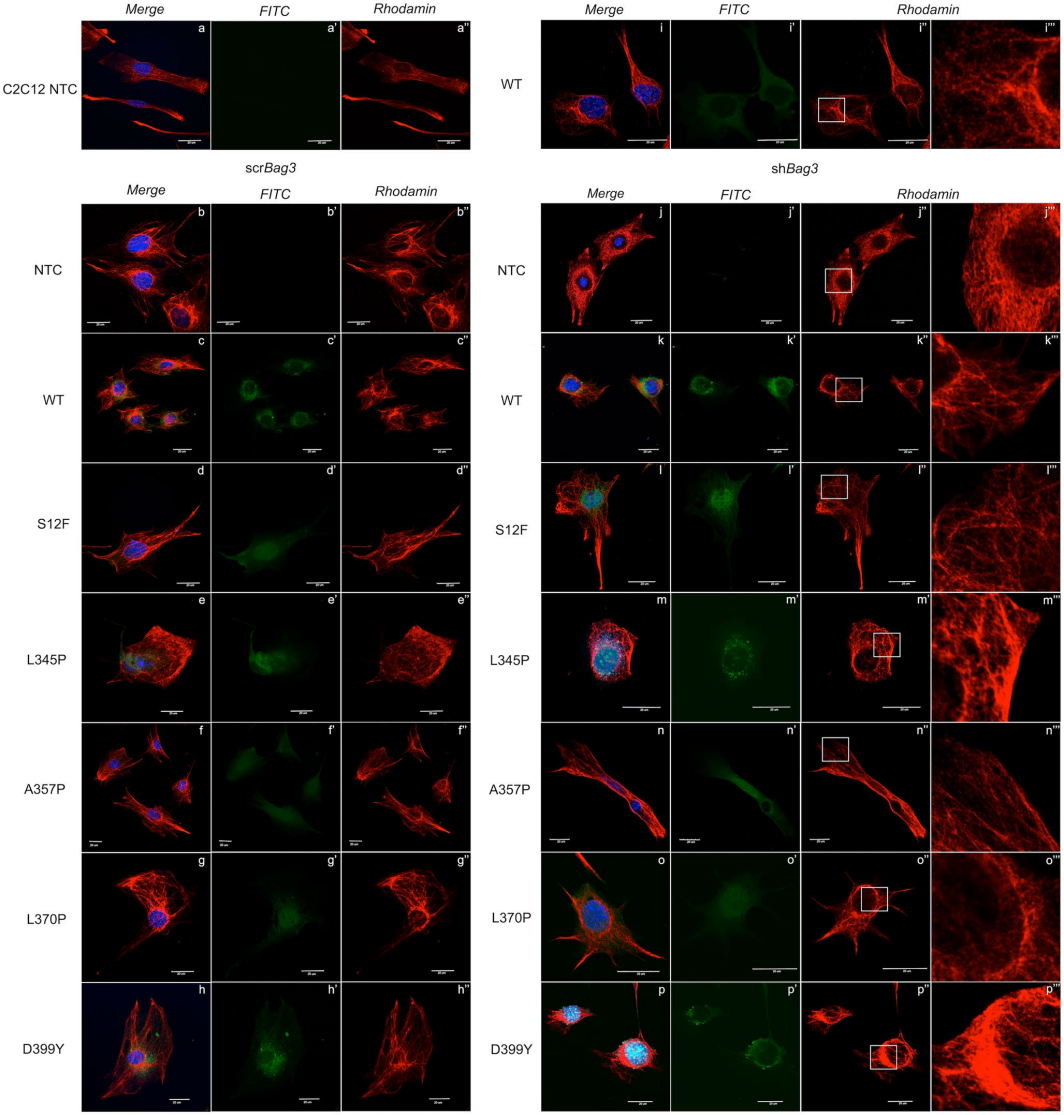
Immunocytochemistry staining for desmin and visualization of desmin aggregates after *Bag3* suppression with sh*Bag3* construct in NTC C2C12 cells and C2C12 transduced with *Des*WT, *Des*S12F, *Des*L345P, *Des*A357P, *Des*L370P, *Des*D399Y lentiviral constructs. (**a**, **a′**, **a″**) Immunofluorescence micrographs of NTC C2C12 cells. (**b**, **b′**, **b″**, **c**, **c′**, **c″**, **d**, **d′**, **d″**, **e**, **e′**, **e″**, **f**, **f′**, **f″**, **g**, **g′**, **g″**, **h**, **h′**, **h″**) Immunofluorescence micrographs of C2C12 with *Des* mutations: *Des*WT, *Des*S12F, *Des*L345P, *Des*A357P, *Des*L370P, *Des*D399Y transduced with scr*Bag3* constructs. (**i**, **i′**, **i″**) Immunofluorescence micrographs of C2C12 transduced with *Des*WT. (**j**, **j′**, **j″**, **j″′**, **k**, **k′**, **k″**, **k″′**, **l**, **l′**, **l″**, **l″′**, **m**, **m′**, **m″**, **m″′**, **n**, **n′**, **n″**, **n″′**, **o**, **o′**, **o″**, **o″′**, **p**, **p′**, **p″**, **p″′**) Immunofluorescence micrographs of C2C12 with various *Des* mutations: *Des*WT, *Des*S12F, *Des*L345P, *Des*A357P, *Des*L370P, *Des*D399Y transduced with sh*Bag3* constructs. (**j″′**, **k″′**, **l″′**, **m″′**, **n″′**, **o″′**, **p″′**) A zoomed area of accumulated desmin aggregates. Cells were immunostained for desmin (Des, red), nuclei DAPI (DAPI, blue), control of LV transduction with GFP (LV, green). Accumulated aggregates are indicated by white boxes

To further prove the role of autophagy in the molecular pathogenesis of desmin-related muscle pathology and aggregate formation, in particular, we analyzed the effect of various *Des* mutations under the suppression of one of the key CASA-related genes—*Bag3* using sh*Bag3* approach. Scr*Bag3* with empty vector used as a control of the possible unwanted effects of LV transduction. We analyzed the formation of desmin aggregates after transduction of C2C12 cells with *Des*WT, *Des*S12F, *Des*L357P, *Des*L345P, and *Des*D399Y on a scr*Bag3* and sh*Bag3* background (Fig. 6). Immunostaining using anti-desmin antibody revealed the three-dimensional desmin filament network in control sh*Bag3*-NTC cells and cells with scr*Bag3* and sh*Bag3* background transduced with *Des*WT, *Des*S12F, *Des*A357P (Fig. 6(j, j′, j″, j″′), (c, c′, c″), (d, d′, d″), (f, f′, f″), (k, k′, k″, k″′), (l, l′, l″, l″′), (n, n′, n″, n″′)). In sh*Bag3*-*Des*L345P (Fig. 6(m, m′, m″, m″′)) and sh*Bag3*-*Des*D399Y cells (Fig. 6(p, p′, p″, p″′)), abnormalities of the desmin filament network and the presence of desmin aggregates in C2C12 were found, while in scr*Bag3*-*Des*L345P (Fig. 6(e, e′, e″)) and scr*Bag3*-*Des*D399Y cells (Fig. 6(h, h′, h″)), the desmin filament network was present. In sh*Bag3*-*Des*L370P cells (Fig. 6(o, o′, o″)), desmin aggregates were not detected; however, the presented filamentous network differed from those in scr*Bag3*-*Des*L370P cells (Fig. 6(g, g′, g″)) and was not presented as solid filaments.

Therefore, the suppression of the CASA pathway resulted in the impairment of desmin filamentous network with the most remarkable changes in *Des*L345P, *Des*L370P, and *Des*D399Y as determined by Western blotting and RNA sequencing. Knock-down of *Bag3* caused the formation of aggregates in the most deleterious *Des* mutations: *Des*L345P and *Des*D399Y.

### *Bag3* knock-down reveals new possible players in maintenance of *Bag3* desmin network stability in C2C12 cells

**Fig. 7:**
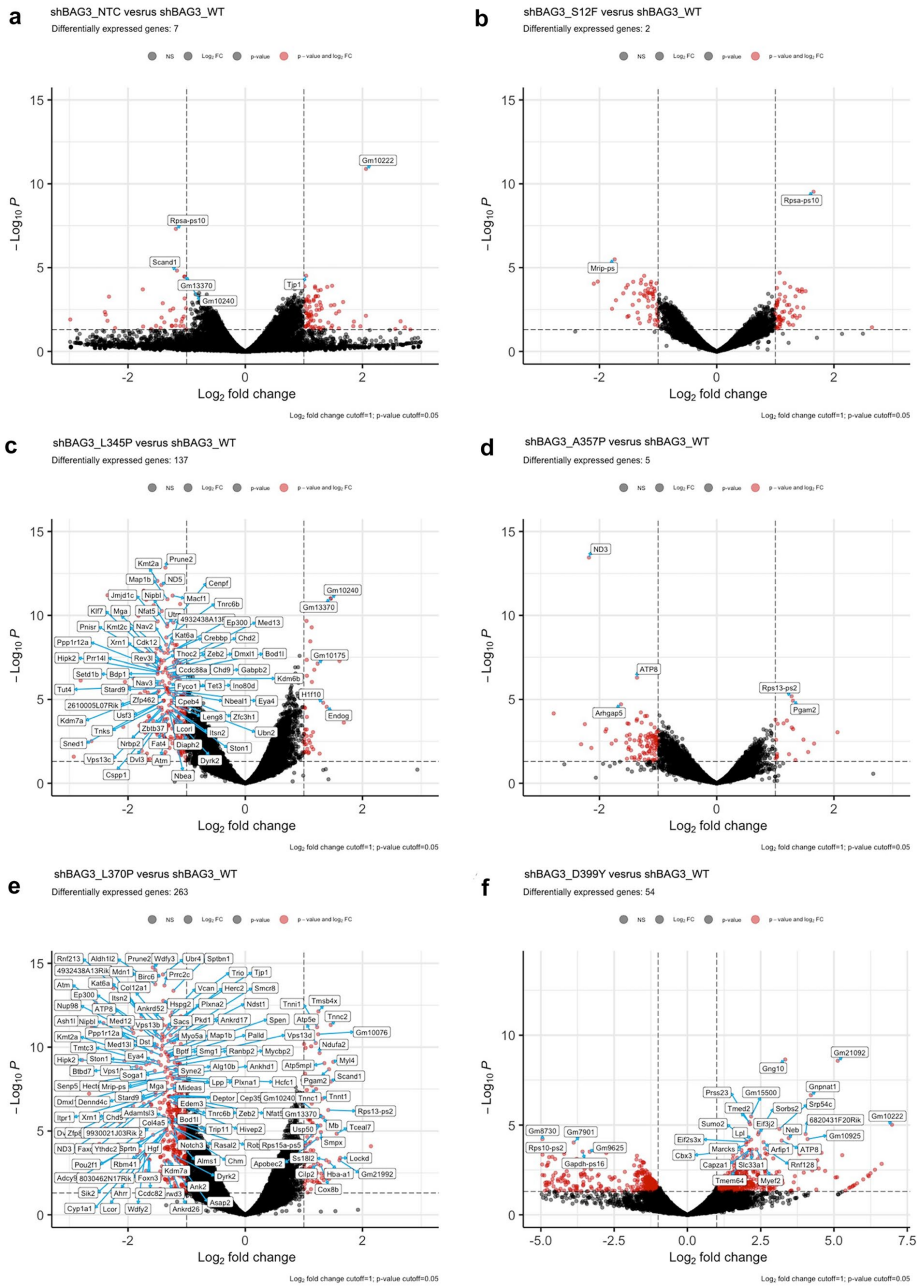
Gene expression analysis by RNA sequencing of C2C12 samples transdused with *Des* mutations: S12F, L345P. A357P, L370P, D399Y on sh*BAG3* background. (**a**, **b**, **c**, **d**, **e**, **f**) Volcano plot illustrations RNA-seq differential expression data. Pairwise comparisons is shown between *Des*WT and the rest of mutations (S12F, L345P, A357P, L370P, and D399Y) on sh*Bag3* background

**Fig. 8:**
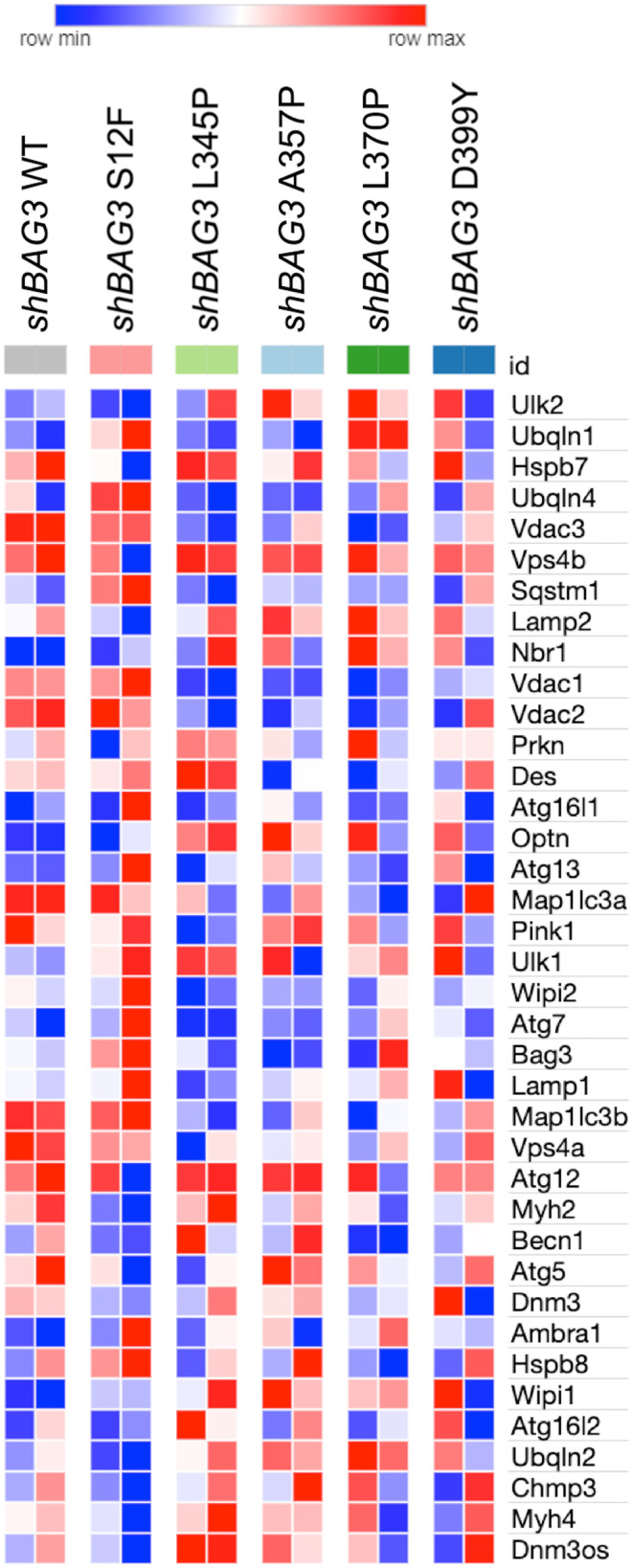
Gene expression analysis by RNA sequencing of C2C12 samples transdused with *Des* mutations: S12F, L345P. A357P, L370P, D399Y on sh*BAG3* background. Heat map illustrating genes associated with autophagy process. Pairwise comparisons is shown between *Des*WT with the rest of mutations (S12F, L345P, A357P, L370P, and D399Y) on sh*Bag3* background. Blue, negative log fold-change (log FC) indicates lower expression; red, positive log FC

To uncover the intracellular processes that determine the pathological effect of *Des* mutations and lead to accumulation of desmin aggregates in *Bag3*-deficient C2C12 cells, transcriptome analysis was performed. A comparison of sh*Bag3* and scr*Bag3* samples revealed that *Bag3*, *Ubqln1*, and *Ubqln4* were downregulated and *Hspb8*, *Hspb7*, *Wipi1*, and *Becn1* were upregulated in sh*Bag3* cells (Supplementary Fig. 3). These findings illustrated that introduction of *shBag3* altered the autophagy pathway. In the next step, we focused on RNA sequencing analysis of *shBag3*-*Des*WT cells compared to and sh*Bag3* cells transduced with *Des* mutations: *Des*S12F, *Des*L345P, *Des*A357P, *Des*L370P, *Des*D399Y. We identified the amount of differentially expressed genes after transduction with different mutations with the maximum number of genes identified in *Des*L370P and *Des*L345P cells (Fig. 7e, c). However, we did not reveal any autophagy pathways that were differentially regulated under the effect of sh*Bag3* and desmin mutations. To analyze further the autophagy- and CASA-related genes in *Bag3-*deficient cells expressing *Des* mutations, we generated heat map visualizations of normalized counts of genes associated with the autophagy process in muscle cells. In most of the studied mutations, we revealed downregulation of the most well-known autophagy genes, such as *Atg16l1*, *Atg7*, *Lamp1*, *Vps4a*, *Map1lc3a*, and *Map1lc3b* (Fig. 8). These data are consistent with previous results; the major differences were observed in more deleterious and aggregate-prone mutations such as *Des*L345P and *Des*L370P, *Des*D399Y.

**Fig. 9:**
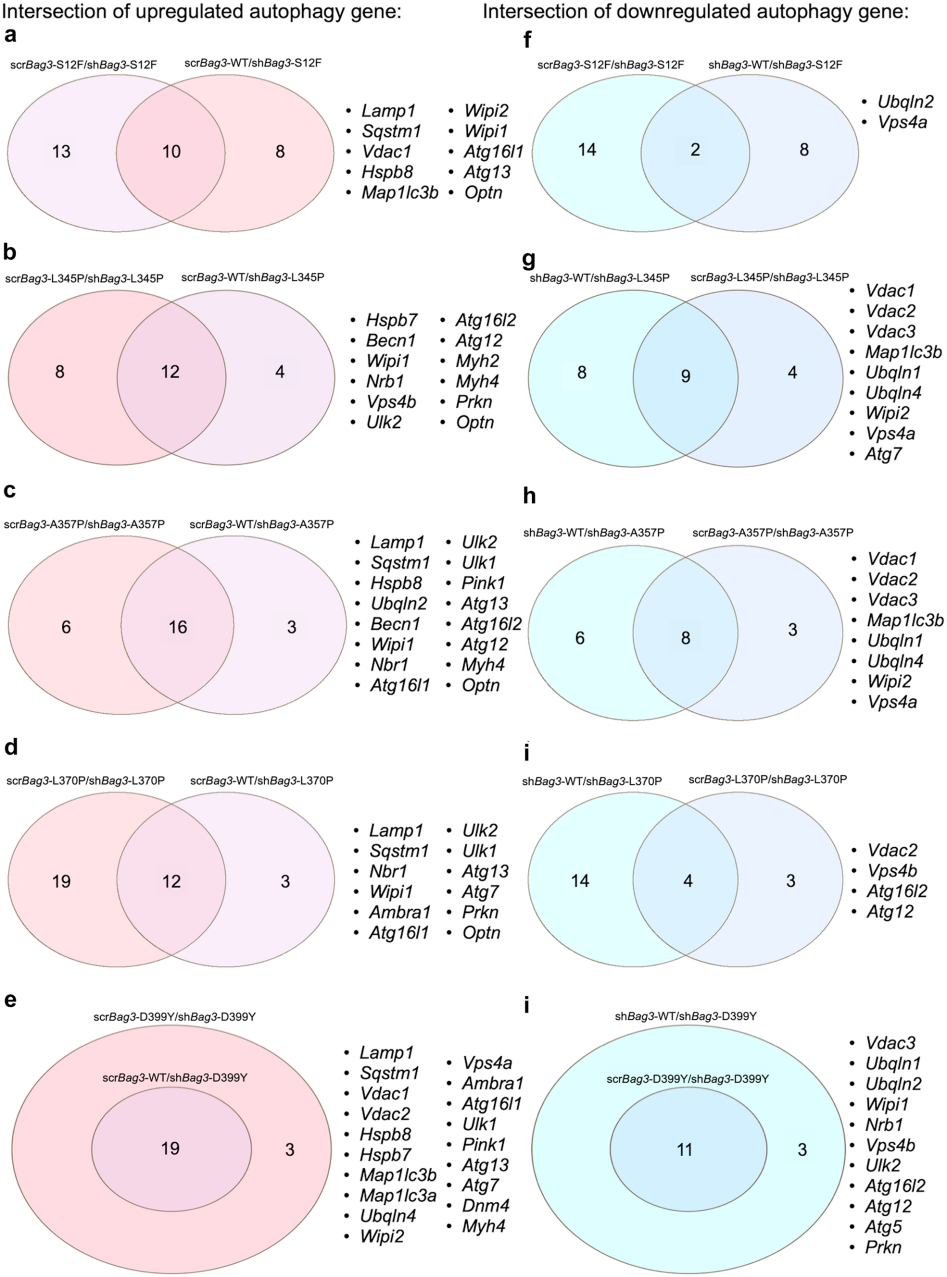
Venn diagram illustration of up- and downregulated overlapping genes between (**a**, **b**, **c**, **d**, **e**) scr*Bag3-Des*Mut/sh*Bag3-Des*Mut comparison and (**f**, **g**, **h**, **i**, **j**) sh*Bag3-Des*WT/sh*Bag3-Des*Mut comparison

Next, we compared each *Des* mutation on scr*Bag3* background to the same mutation on sh*Bag3* background to identify the up- and downregulated autophagy-related gene sets and estimate a potential effect of *Bag3* knock-down on *Des* mutation phenotype. We also compared the effect of *Des* mutations on sh*Bag3* background with sh*Bag3*-*Des*WT to identify the up- and downregulated autophagy-related gene sets and estimate a potential effect of a specific *Des* mutation under *Bag3* downregulation background. For each *Des* mutation pair, we compared both up- and downregulated gene sets, respectively, using the Venn diagram, which allowed us to pick up the genes both affected by *Des* mutations and *Bag3* knock-down and potentially linked to the aggregate formation (Fig. 9(a–j)). This comparison allowed us to list *Ulk2*, *Hspb7*, *Hspb8*, *Wipi2*, *Pink*, and *Atg16l1* as most commonly upregulated and *Vdac1*, *Vdac2*, *Vdac3*, *Myh2*, and *Myh4* as most commonly downregulated genes. The obtained RNA sequencing results were partially confirmed by qPCR (Supplementary Fig. 4).

The overlap of the obtained gene sets with the genes identified by the heat map in scr*Bag3* cells confirmed the important role of autophagy in the mitigation of *Des* mutations effects by degrading intracellular desmin aggregates. The identification of new genes in sh*Bag3* samples revealed additional possible players: *Ulk2*, *Hspb7*, *Hspb8*, *Wipi2*, *Pink*, *Atg16l1*, *Vdac1*, *Vdac2*, *Vdac3*, *Myh2*, *Myh4*, involved in the process of desmin aggregates accumulation in cells with *Des*L345P, *Des*L370P, and *Des*D399Y mutations.

## Discussion

*DES* mutations have been linked to the development of neuromuscular and cardiac muscle pathologies for over two decades ((Goebel 1995); (Goldfarb et al. 1998); (Munoz-Marmol et al. 1998)). On the cellular level, the disruption of muscle fibers and mutation aggregation propensity play a key role in the disease progression. It is well known that the autophagy process eliminates cytosolic protein aggregates and is essential for protein quality control under stressful conditions (Klionsky and Emr 2000). In highly dynamic systems, such as muscle and cardiac tissues, where the turnover of proteins normally is at very high level due to mechanotransduction, the autophagy machinery needs to be perfectly functional (Millward et al. 1975) (Tipton et al. 2018). The mechanisms of an autophagy flux arrest, bringing to the development of severe muscle pathologies, are being actively studied. In the three-dimensional intracellular space of the muscle fibers, desmin, apart from its structural functions, acts as a transport network during the autophagosome maturation process and their fusion with lysosomes ((Capetanaki et al. 2007); (Tsoupri and Capetanaki 2013)). Therefore, it is reasonable to consider the alteration of the autophagy process as one of the key molecular mechanisms leading to desmin-related pathologies ((Capetanaki et al. 2015); (Huang et al. 2021)).

In the our studies, we described a regular turnover of LC3-I to LC3-II in NTC and *Des*WT C2C12 cells during autophagy induction to characterize the autophagy flux in muscle cells (Sukhareva et al. 2021). The level of LC3-I steadily declined with the time of nutritional stress, while the level of LC3-II did not change or only slightly increased at the beginning and then substantially degraded to the later time points. Notably, the observed autophagy dynamics differed from autophagy dynamics in HeLa, MEF, and HEK293, where the major accumulation of LC3-II was observed in the early autophagy stages (Klionsky et al. 2016). This difference can reflect a higher rate of autophagy turnover in muscle cells required for muscle protein homeostasis (Sebastian and Zorzano 2020). Moreover, in all samples after LV transduction, we did not observe LC3-II accumulation with time of autophagy stimulation even though basal LC3-II levels were increased before the induction of starvation. Thus, in *Des*L345P samples, basal LC3-II level was increased, which can be a sign of the basal autophagy induction in response to aggregate-prone *Des* mutation, whereas non-aggregate mutations did not show any significant LC3-II accumulation. In this sense, the decline in LC3-I content without autophagy induction represented conversion to the LC3-II.

The induction of autophagy by 2 h of serum deprivation revealed that in *Des*L345P, LC3-I protein amount declined faster than in *Des*WT, which can signify a high autophagosome formation rate. On the other hand, in *Des*L370P and *Des*D399Y, the conversion of LC3-I was slower, suggesting an impairment in the autophagosome maturation process. Consequently, the autophagosome degradation for some mutations was more intensive, such as *Des*L370P and *Des*D399Y mutations, where the LC3-II level fell below *Des*WT, while in *Des*L345P LC3-II level fell behind *Des*WT. In addition, in *Des*L345P cells, LC3-I conversion to LC3-II was not compensated, possibly due to the loss of LC3-II degradation capacity and rapid turnover. *Des*L370P and *Des*D399Y mutations demonstrated a similar effect on the autophagy process. In both mutations, the deceleration of LC3-I conversion and a considerable decline in LC3-II level compared to *Des*WT were observed. These findings would seem to suggest either an alteration of autophagosome maturation or a higher autophagosome degradation rate.

There are several potential explanations for the observed autophagy dynamics. First, autophagy can be hampered by the disruption of desmin filaments serving as a transport path for autophagosome vesicle maturation and its fusion with lysosome. Second, the increased of autophagy flux and degradation rate can occur as a response to the presence of protein aggregates (Winter et al. 2019). To test which of two plausible mechanisms is involved, the inhibitor of lysosome and autophagosome fusion stage CQ was applied in the subsequent experiments.

In *Des*L345P, the effect of CQ was depicted by a massive accumulation of LC3-II and the reduction of LC3-I after 2 h of starvation. Similar dynamics occurred in *Des*D399Y. Thus, we suggested that both mutations induced a higher rate of autophagosome formation. Similar autophagy dynamics for *Des*D399Y mutation was shown in a study made by Lorena (Perrone et al. 2019). However, the difference in LC3-I after 2 h of starvation in the presence of CQ in *Des*L345P and *Des*D399Y indicated that for *Des*L345P the degradation process was mainly disrupted, while in *Des*D399Y both degradation and recycling proceeded faster compared to *Des*WT.

Looking into the protein dynamics of *Des*L370P after 2 h of starvation under the effect of CQ, we observed LC3-II was not accumulated compared to *Des*WT. Hence, we concluded that in *Des*L370P samples, the conversion of LC3-I to LC3-II was impaired, suggesting that the autophagosomes did not form properly even though the degradation and recycling processes proceeded normally.

**Fig. 10:**
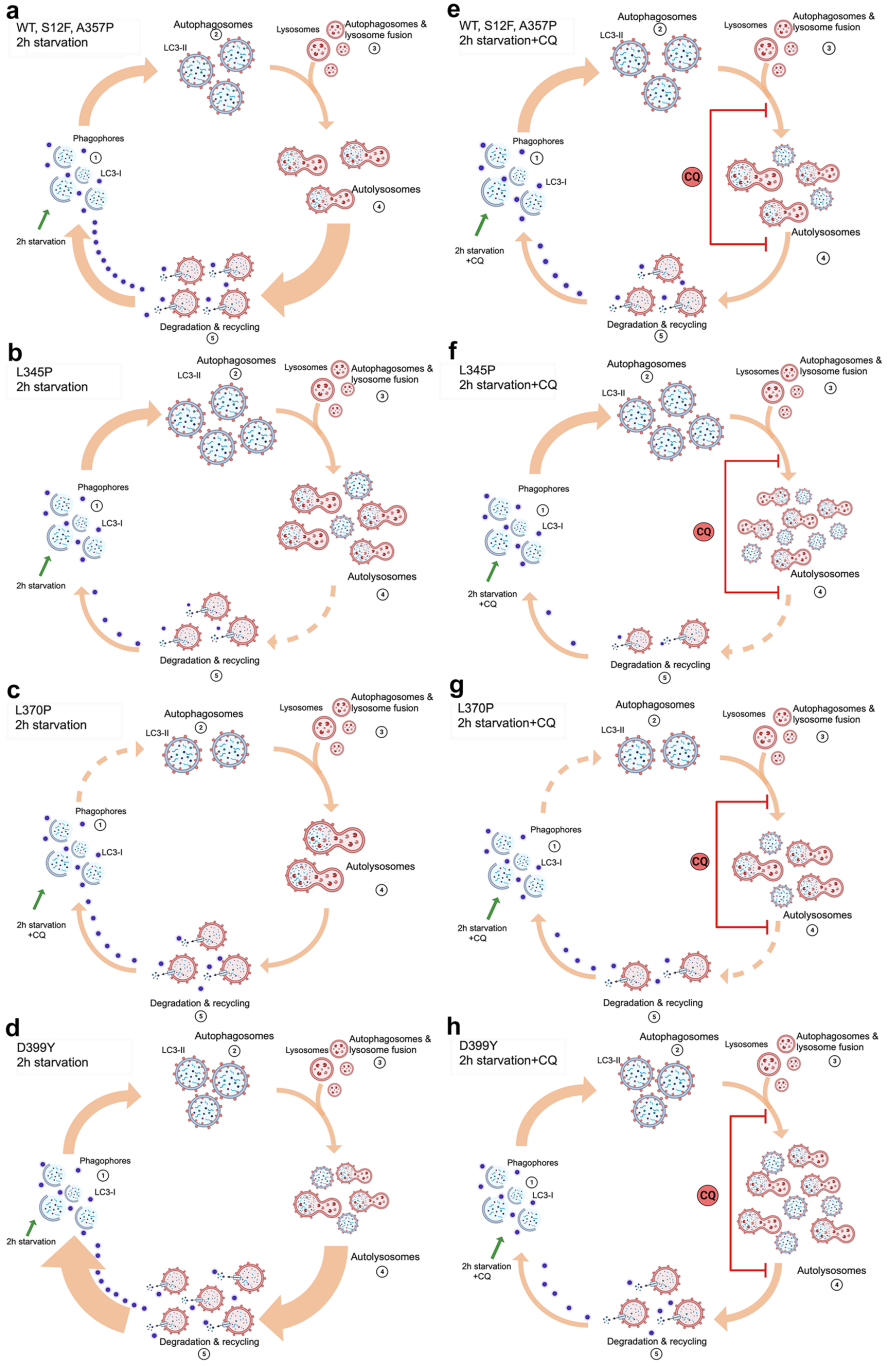
Proposed mechanisms of *Des* mutations effect on autophagy pathway. Created via BioRender. The scheme illustrated the alterations in normal conditions (**a**, **b**, **c**, **d**) and after the chloroquine (CQ) blockage (**e**, **f**, **g**, **h**). Several autophagy patterns influenced by *Des* mutations are illustrated. (**a**, **b**) *Des*WT, *Des*S12F, and *Des*A357P. (**b**, **f**) for *Des*L345P. (**c**, **g**) *Des*L370P. (**d**, **h**) *Des*D399Y. Autophagy dynamics (**a**, **e**) for *Des*WT, *Des*S12F, and *Des*A357P demonstrates the normal protein turnover patter in muscle cells with increased rate of autophagosomes formation, its subsequent fusion with lysosomes and degradation. (**b**, **f**) *Des*L345P results in violation of autolysosomes degradation process; therefore, the partial return of LC3-II to LC3-I is detained. (**c**, **g**) In *Des*L370P, the process of autophagosome maturation is impaired due to delay in the formation of autophagosomes. (**d**, **h**) *Des*D399Y demonstrates the increased turnover speed with the highest autophagosome formation and degradation rate

To summarize, all studied *Des* mutations showed an effect on autophagy flux in muscle C2C12 cells with either a predominant impact on autophagosome maturation or on degradation and recycling processes. As a result, we proposed the following model of autophagy flux in the presence of *Des* mutations (Fig. 10a–h). Earlier, Bar et al. and Kreplak et al. demonstrated that proline mutations in *DES* could cause a severe disruption of desmin filament assembly at various stages of polymerization leading to the different mechanical properties of desmin filaments ((Wawersik et al. 1997); (Bar et al. 2005); (Kreplak and Bar 2009)). Given that the filament assembly for these mutations was altered at different stages, we assumed that the abnormal desmin polymerization process could deteriorate various protein-protein interactions. For this reason, the impairment of proteostasis and decreased autophagy performance in the presence of *Des* mutations took place at distinct steps of the autophagy process: either deteriorating or, as for *Des*D399Y mutation, accelerating the autophagy flux to support homeostasis.

In line with Western blotting data, RNA sequence demonstrated no difference in autophagy-related gene expression between *Des*WT and cells carrying *Des*S12F, *Des*A357P. The major effect and the highest number of differentially expressed genes were determined in *Des*L345P, *Des*L370P, and *Des*D399Y samples. Thus, *Des*L345P cells where significant increase in the level of LC3-II at the basal level was observed by Western Blotting, demonstrated the upregulation of autophagy-related genes, such as *Map1lc3a*, *Map1lc3b*, *Becn1*, and *Sqstm1* compared to *Des*WT. Therefore, we consider that basal upregulation of autophagy flux in muscle cells initially serves to prevent the accumulation of mutant desmin in cells and desmin aggregate formation.

To further delineate the role of autophagy in aggregate formation, we suppressed the expression of a key effector protein in chaperone-mediated autophagy *Bag3* using sh*Bag3* approach. Consequently, the basal autophagy flux was arrested. The *Bag3* knock-down allowed us to visualize the desmin aggregates in *Des*L345P, *Des*D399Y, and *Des*L370P cells which were not previously seen in native or scr*Bag3* background. In contrast, in *Des*S12F and *Des*A357P cells, where the significant difference in autophagy efficiency was not detected, the filament networks remained detectable in all samples with sh*Bag3* and *Des* mutations. Therefore, the disruption of a desmin filament network and the presence of protein aggregates caused by autophagy restriction stressed the role of autophagy in the elimination of mutant desmin aggregates from cells. Sh*Bag3* samples analyzed by RNA sequencing further confirmed the highest effect of *Des*L345P, *Des*L370P, and *Des*D399Y mutations. Most of differentially expressed genes were detected in the samples where aggregates were formed. Thus, RNA sequencing data well corresponded to Western blotting and immunocytocheminstry results showing the most prominent effect on autophagy of *Des*L345, *Des*L370P, and *Des*D399Y mutations, where the most common downregulated genes on sh*Bag3* background were *Vdac2* and *Vps4a*. The contribution of autophagy-related genes in aggregate accumulation is still under discussion because of seeming contradictions in available data ((Malta et al. 2019); (Pan et al. 2019)). Autophagy is a highly conserved and diversified process. For this reason, if some of the autophagy pathways are blocked, others stay active or become upregulated. Even though, in the presented study, we suppressed some autophagy paths by *Bag3* knock-down or by introduction of *Des* mutations, or at some point both, it does obligatory lead to an ultimate and total block the entire autophagy process (Sandri 2010). Since autophagy pathway is very crucial for muscle cell viability, when suppressing one branch of autophagy pathway the other can activate more intensively, leading to a temporal compensation. Thus, the detailed delineation of exact mechanisms of autophagy impairment in various desmin mutations can help the precise targeting of molecular pathogenesis of desmin-related myopathies and cardiomyopathies.

## Conclusion

In the current study, we have focused on the autophagy impairment in muscle cells under the effect of various *Des* mutations using Western blotting, immunocytochemistry, RNA sequencing analysis, and shRNA approach. We showed that for studied *Des* mutations, stress stimulation of autophagy affected the autophagy flux in a mutation-specific manner. We demonstrated that *Des* mutations are often associated with an increased basal autophagy rate and upregulation of genes responsible for autophagy-mediated protein degradation. The latter phenomenon, possibly, can represent a basis for a long-time compensation of desmin aggregate formation in muscle cells regardless of the presence of mutant desmin filaments. We showed that suppression of *Bag3* gene with sh*Bag3* impaired autophagy turnover, resulting in more prominent disruption of desmin filament network promoting the aggregates accumulation. Further, we identified several autophagy-related genes which we suggest are associated with maintaining aggregate-free cellular status of muscle cells for an extended period. Thus, this study can be a good starting point for further research on the autophagy process in muscle cells with pathological *Des* mutations linked to severe cases of myopathies.

## Limitations

SNormaleveral limitations of the current study restrict the full interpretation of the obtained results due to some technical caveats. Thus, *lentiviral transduction* of C2C12 with *Des* constructs caused a toxic effect that was found in all performed experiments, and a comparison of NTC and *Des*WT detected that viral transduction itself induced LC3-I conversion. The number of experiments carried out for *Western blotting* analysis was limited to six and the number of *immunocytochemistry* experiments to only three. In each experiment, the number of cells counted for each mutation and each time point was 50. The partial incoherence of immunocytochemistry and Western blotting results could potentially be explained by the morphological difference of autophagosomes among different *Des* mutations. For example, in *Des*S12F, the autophagosome number could be greater than in *Des*WT, but their size was smaller. Conversely, in comparison of *Des*L345P and *Des*WT, where the difference in autophagosome number was absent, it could be because of the bigger autophagosome size in *Des*L345P. Unfortunately, it was impossible to perform *immunocytochemistry* staining for samples under the effect of CQ due to the low viability of cells and decrease in cell adherence throughout the experiment (Supplementary Fig. 5). In addition, for *RNA sequencing*, all samples were analyzed in duplicates and autophagy dynamics on samples with sh*Bag3* background was not evaluated by Western blotting due to the low viability of cells in the presence of severe *Des* mutations. The above technical and design caveats can limit the extrapolation of the data obtained.

## Supplementary Information

Below is the link to the electronic supplementary material.

Supplementary Figure 1. Autophagy dynamics in NTC C2C12 cells and C2C12 carrying various *Des* mutations (*Des*WT, *Des*S12F, *Des*L345P, *Des*A357P, *Des*L370P, *Des*D399Y) illustrated by the relative numbers of autophagosome in basal conditions and after 2h of starvation. (a) - Graph representation of relative autophagosome number (a’) in basal conditions and (a’’) after 2h of autophagy stimulation. (b) - Immunofluorescence micrographs of autophagosomes in NTC C2C12 cells and C2C12 with various *Des* mutations *Des*WT, *Des*S12F, *Des*L345P,* Des*A357P, *Des*L370P, *Des*D399Y stained for LC3-II in control samples and samples after 2h of serum deprivation. Cells were immunostained for LC3 (LC3, red) and nuclei DAPI (DAPI, blue). x100, * < 0.05. Data presented as mean + - SD. n = 50 counted cells. (TIFF 22678 KB)

Supplementary Figure 2. Gene expression by qPCR in NTC C2C12 cells and C2C12 cells transduced with *Des*WT, *Des*S12F, *Des*L345P, *Des*A357P, *Des*L370P, *Des*D399Y lentivaral constructions. (a) - Transcript levels of *Des* compared by real-time PCR analysis between NTC C2C12 and mutant samples. (b) - Transcript levels of *Sqstm* compared by real-time PCR analysis between NTC C2C12 and mutant samples. (c) - Transcript levels of *Bag3* compared by real-time PCR analysis between NTC C2C12 and mutant samples. (d) - Transcript levels of *Hspb7 *compared by real-time PCR analysis. (e) - Transcript levels of *Hspb8* compared by real-time PCR analysis. * < 0.05. Data presented as mean + - SD. n = 3 biological replicates. (TIFF 33414 KB)

Supplementary Fig. 3. Gene expression analysis by RNA sequencing of C2C12 samples transuded with *Des* mutations: S12F, L345P. A357P, L370P, D399Y on shBag3 and scrBag3 background. (a)—Volcano plot illustration of RNA-seq differential expression data. Pairwise comparisons is shown for each desmin transduction between scr*Bag3* and sh*Bag3* background. (b) Heat map is illustrating genes associated with autophagy process. Pairwise comparisons is shown between all DesMut samples on scr*Bag3* background and all DesMut samples on sh*Bag3* background. Blue, negative log fold-change (log FC) indicates lower expression; red, positive log FC. (TIFF 41250 KB)

Supplementary Fig. 4. Gene expression by qPCR of C2C12 samples transuded with *Des* mutations: S12F, L345P. A357P, L370P, D399Y on sh*Bag3* background. (a)—Transcript levels of *Sqstm* compared by real-time PCR analysis between *Des*WT and mutant samples shBag3 background. (b)—Transcript levels of* Vdac2* compared by real-time PCR analysis between *Des*WT and mutant samples sh*Bag3* background. (c)—Transcript levels of Ulk1 compared by real-time PCR analysis between *Des*WT and mutant samples sh*Bag3 *background. (d)—Transcript levels of *Atg7* compared by real-time PCR analysis between *Des*WT and mutant samples sh*Bag3* background. n = 3 biological replicates. (TIFF 14069 KB)

Supplementary Fig. 5. Cell survival assay. The graph demonstrates the decrease in cell survival after viral transduction with various *Des* mutations, autophagy stimulation for 2 h of starvation and the effect of CQ. Data is presented as a relative amount of alive cells The results were obtained using propidium iodide (PI) dye by flow cytometry. (TIFF 14020 KB)

Supplementary Table 1. Primers sequences complementary for mouse used for qPCR analysis. (JPG 146874 KB)
