# Supplementary figures and images for: Desmin mutations impact the autophagy flux in C2C12 cell in mutation-specific manner
Source: Cell Tissue Res. 2023 Jun 6;393(2):357–75. doi: 10.1007/s00441-023-03790-6 (PMC10406715; doi:10.1007/s00441-023-03790-6)

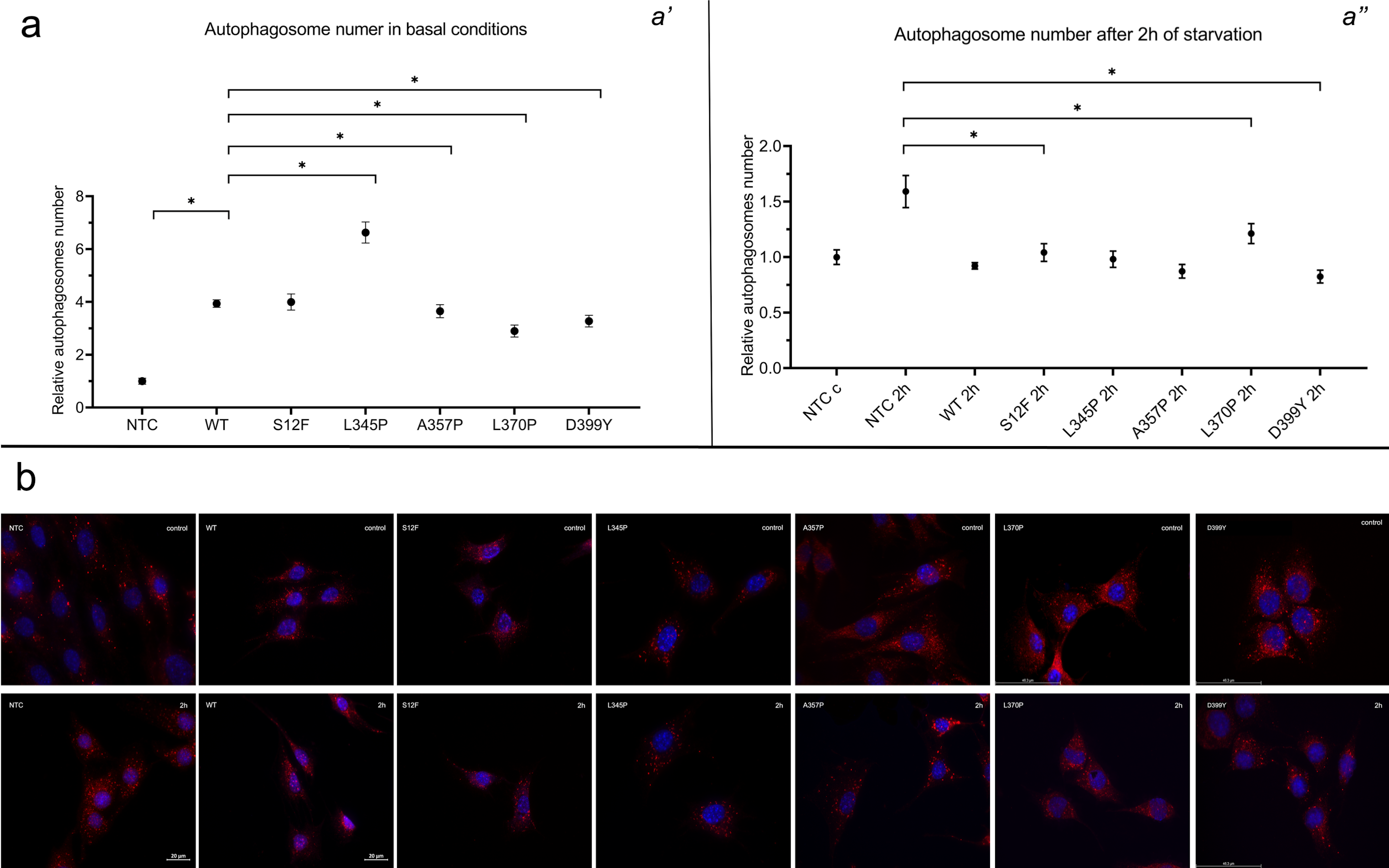

Supplement: Supplementary file 1 — Supplementary Figure 1. Autophagy dynamics in NTC C2C12 cells and C2C12 carrying various Des mutations (DesWT, DesS12F, DesL345P, DesA357P, DesL370P, DesD399Y) illustrated by the relative numbers of autophagosome in basal conditions and after 2h of starvation. (a) - Graph representation of relative autophagosome number (a’) in basal conditions and (a’’) after 2h of autophagy stimulation. (b) - Immunofluorescence micrographs of autophagosomes in NTC C2C12 cells and C2C12 with various Des mutations DesWT, DesS12F, DesL345P, DesA357P, DesL370P, DesD399Y stained for LC3-II in control samples and samples after 2h of serum deprivation. Cells were immunostained for LC3 (LC3, red) and nuclei DAPI (DAPI, blue). x100, * < 0.05. Data presented as mean + - SD. n = 50 counted cells. (TIFF 22678 KB) [file 441_2023_3790_MOESM1_ESM.tiff]

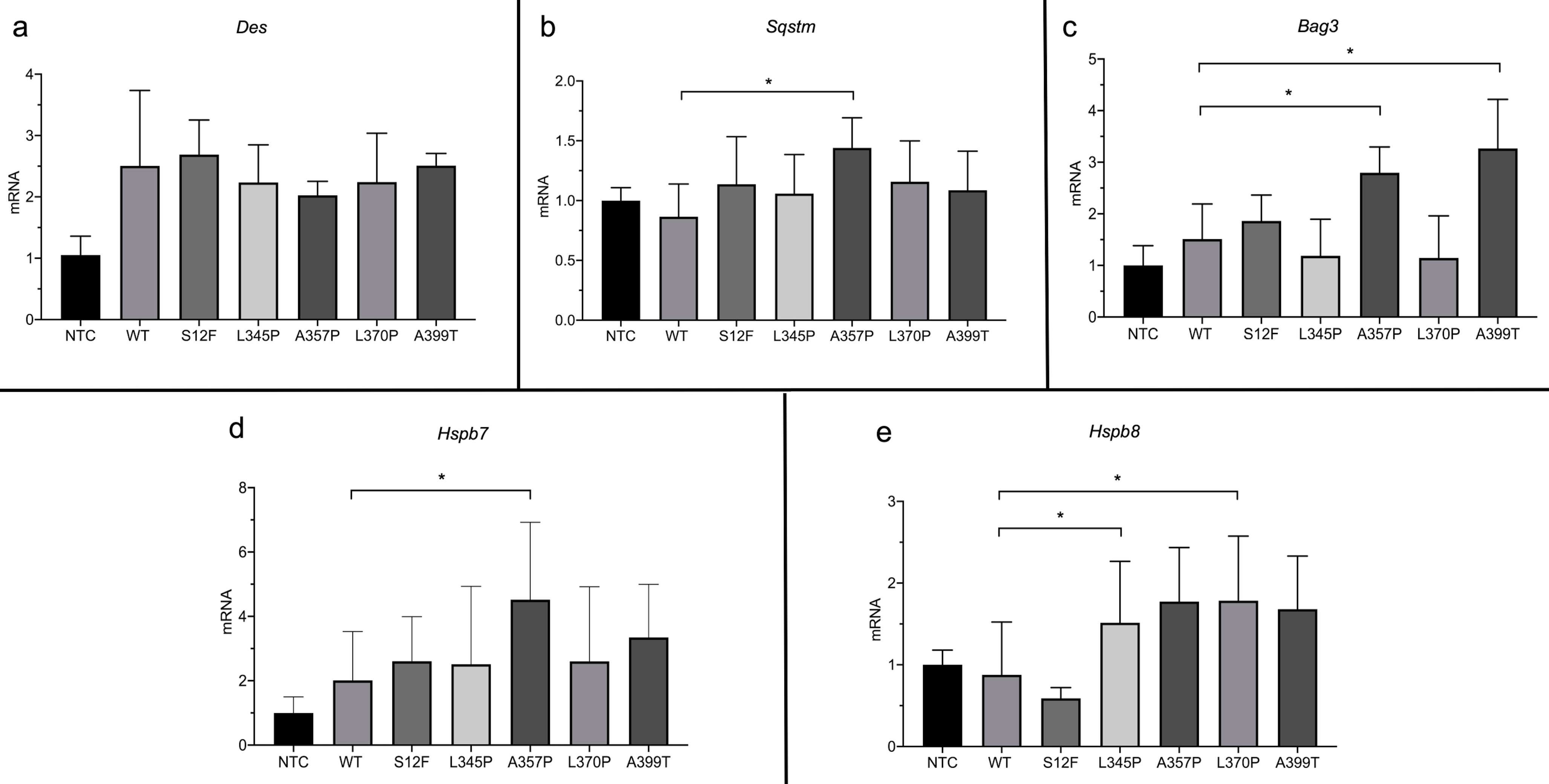

Supplement: Supplementary file 2 — Supplementary Figure 2. Gene expression by qPCR in NTC C2C12 cells and C2C12 cells transduced with DesWT, DesS12F, DesL345P, DesA357P, DesL370P, DesD399Y lentivaral constructions. (a) - Transcript levels of Des compared by real-time PCR analysis between NTC C2C12 and mutant samples. (b) - Transcript levels of Sqstm compared by real-time PCR analysis between NTC C2C12 and mutant samples. (c) - Transcript levels of Bag3 compared by real-time PCR analysis between NTC C2C12 and mutant samples. (d) - Transcript levels of Hspb7 compared by real-time PCR analysis. (e) - Transcript levels of Hspb8 compared by real-time PCR analysis. * < 0.05. Data presented as mean + - SD. n = 3 biological replicates. (TIFF 33414 KB) [file 441_2023_3790_MOESM2_ESM.tiff]

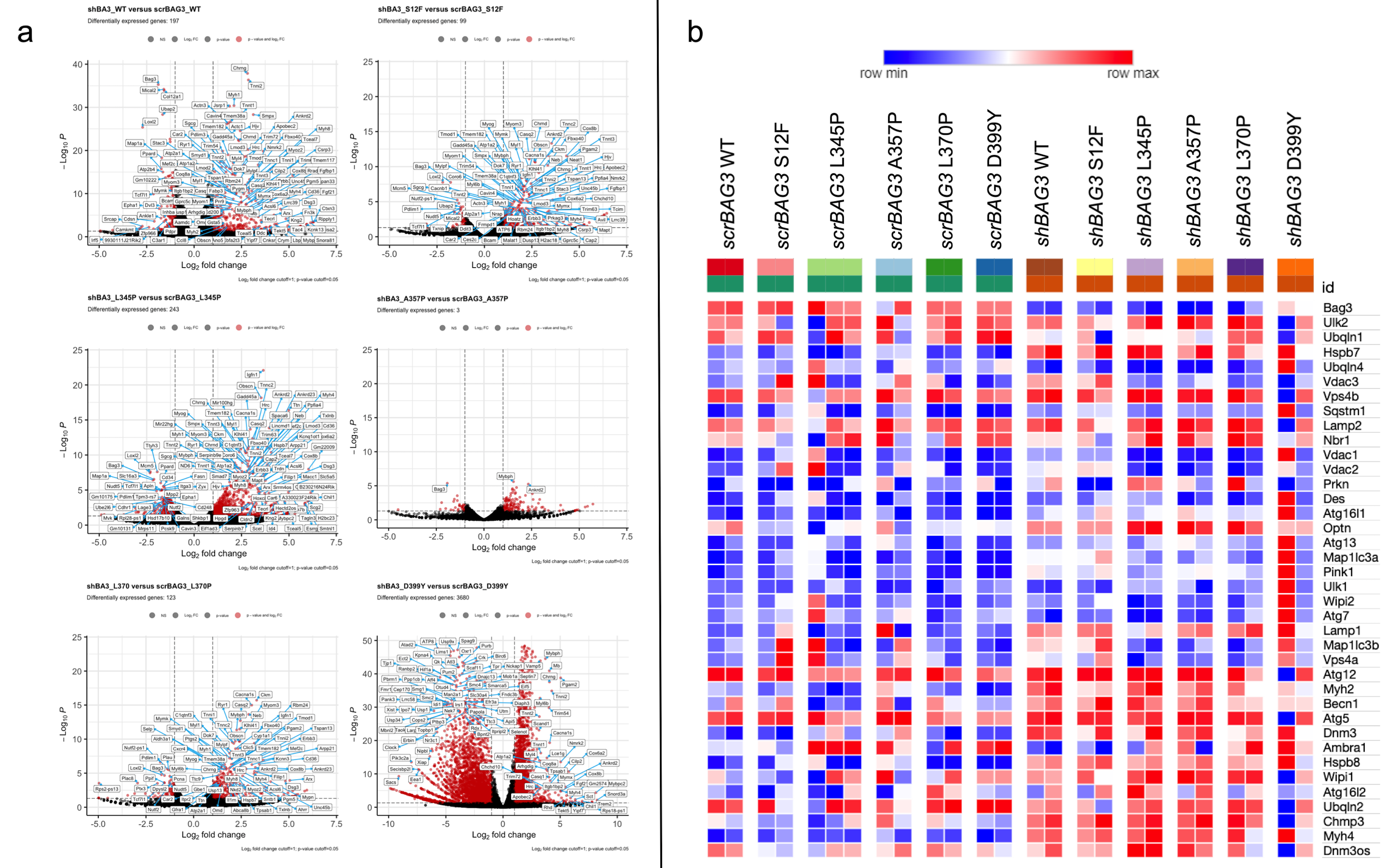

Supplement: Supplementary file 3 — Supplementary Fig. 3. Gene expression analysis by RNA sequencing of C2C12 samples transuded with Des mutations: S12F, L345P. A357P, L370P, D399Y on shBag3 and scrBag3 background. (a)—Volcano plot illustration of RNA-seq differential expression data. Pairwise comparisons is shown for each desmin transduction between scrBag3 and shBag3 background. (b) Heat map is illustrating genes associated with autophagy process. Pairwise comparisons is shown between all DesMut samples on scrBag3 background and all DesMut samples on shBag3 background. Blue, negative log fold-change (log FC) indicates lower expression; red, positive log FC. (TIFF 41250 KB) [file 441_2023_3790_MOESM3_ESM.tiff]

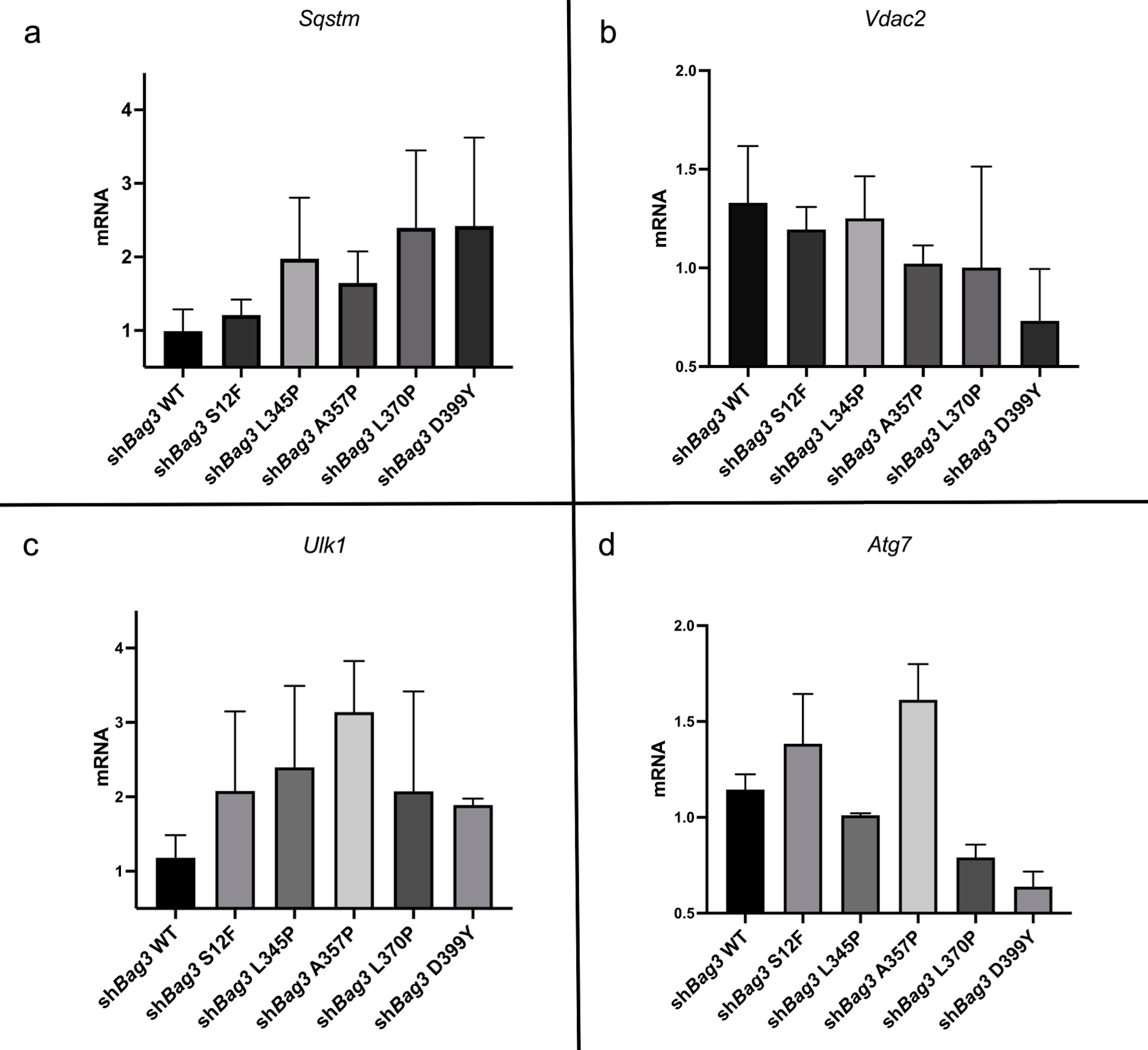

Supplement: Supplementary file 4 — Supplementary Fig. 4. Gene expression by qPCR of C2C12 samples transuded with Des mutations: S12F, L345P. A357P, L370P, D399Y on shBag3 background. (a)—Transcript levels of Sqstm compared by real-time PCR analysis between DesWT and mutant samples shBag3 background. (b)—Transcript levels of Vdac2 compared by real-time PCR analysis between DesWT and mutant samples shBag3 background. (c)—Transcript levels of Ulk1 compared by real-time PCR analysis between DesWT and mutant samples shBag3 background. (d)—Transcript levels of Atg7 compared by real-time PCR analysis between DesWT and mutant samples shBag3 background. n = 3 biological replicates. (TIFF 14069 KB) [file 441_2023_3790_MOESM4_ESM.tiff]

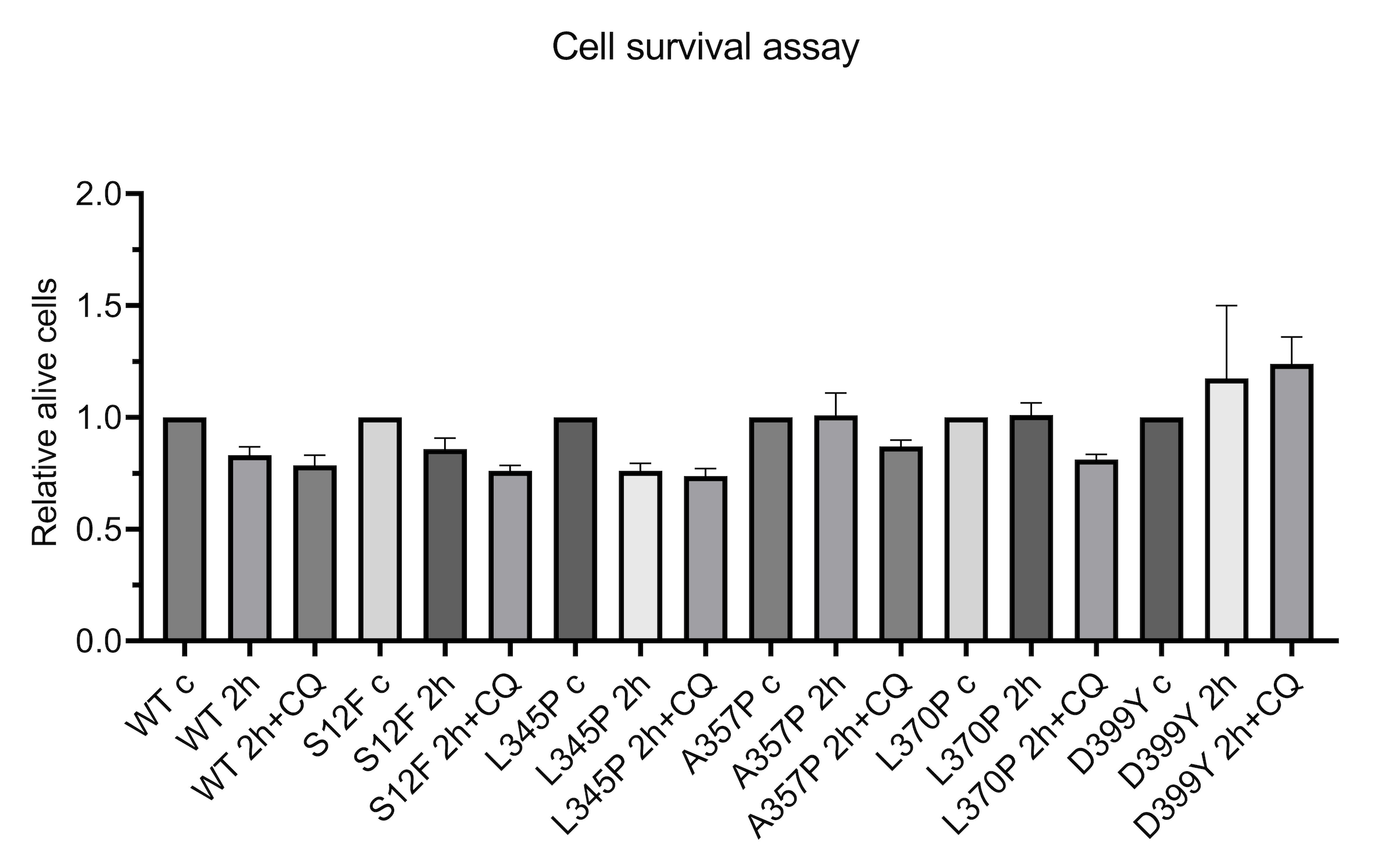

Supplement: Supplementary file 5 — Supplementary Fig. 5. Cell survival assay. The graph demonstrates the decrease in cell survival after viral transduction with various Des mutations, autophagy stimulation for 2 h of starvation and the effect of CQ. Data is presented as a relative amount of alive cells The results were obtained using propidium iodide (PI) dye by flow cytometry. (TIFF 14020 KB) [file 441_2023_3790_MOESM5_ESM.tiff]

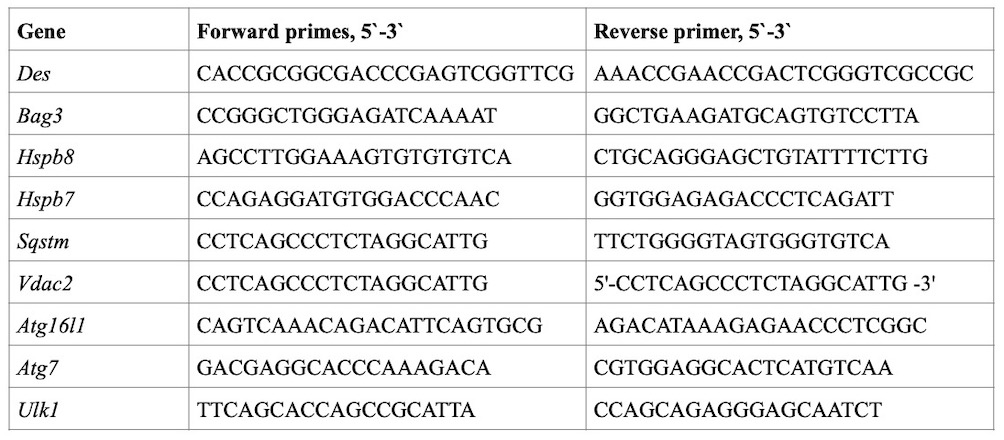

Supplement: Supplementary file 6 — Supplementary Table 1. Primers sequences complementary for mouse used for qPCR analysis. (JPG 146874 KB) [file 441_2023_3790_MOESM6_ESM.jpg]
